# The emerging role of alternative splicing in senescence and aging

**DOI:** 10.1111/acel.12646

**Published:** 2017-07-13

**Authors:** Mathieu Deschênes, Benoit Chabot

**Affiliations:** ^1^ Department of Microbiology and Infectious Diseases Faculty of Medicine and Health Sciences Université de Sherbrooke Sherbrooke Quebec J1E 4K8 Canada

**Keywords:** aging, alternative splicing, pre‐mRNA, RNA, RNA binding proteins, senescence, splice variants, splicing

## Abstract

Deregulation of precursor mRNA splicing is associated with many illnesses and has been linked to age‐related chronic diseases. Here we review recent progress documenting how defects in the machinery that performs intron removal and controls splice site selection contribute to cellular senescence and organismal aging. We discuss the functional association linking *p53, IGF‐1, SIRT1,* and *ING‐1* splice variants with senescence and aging, and review a selection of splicing defects occurring in accelerated aging (progeria), vascular aging, and Alzheimer's disease. Overall, it is becoming increasingly clear that changes in the activity of splicing factors and in the production of key splice variants can impact cellular senescence and the aging phenotype.

## Introduction

**Figure 1:**
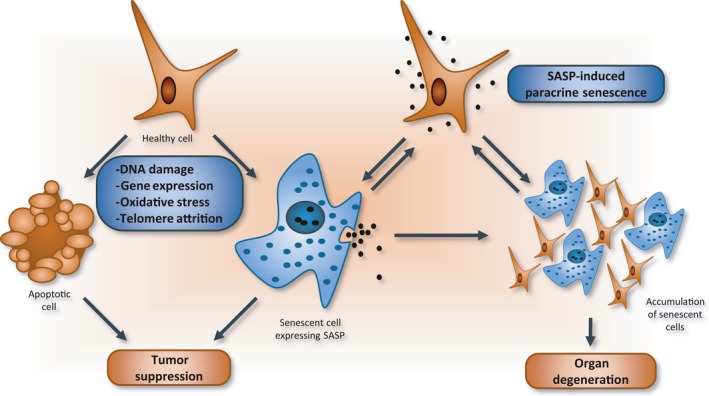
Senescence leads to organ degeneration. The constant exposure of cells to intrinsic or extrinsic stresses may lead to senescence or apoptosis. Senescence‐associated secretory phenotypes (SASP) trigger paracrine senescence in neighboring areas to enhance senescence in tissues. When an aging immune system fails to clear senescent cells, they accumulate in tissues over time, ultimately leading to organ dysfunction.

Aging is defined as a progressive decline of fitness over time, ultimately leading to death (Kirkwood & Holliday, 1979; Kirkwood, 2005). This decline is associated with several changes such as tissue deterioration and disorganization, organ dysfunction, and loss of stem cell renewal, the latter contributing to age‐associated immunodeficiency. At the cellular and molecular levels, the aging phenotype varies between tissues but can include common hallmarks such as genomic and epigenetic instability, mitochondrial dysfunction, telomere attrition, and the accumulation of senescent cells (Kirkwood & Holliday, 1979; Kirkwood, 2005; López‐Otín *et al*., 2013). Considered as one of the causes of age‐related tissue degeneration, cellular senescence is an irreversible and programmed cell‐cycle arrest that occurs in most diploid cell types (Hayflick & Moorhead, 1961). Senescence is associated with large‐scale changes affecting a variety of processes such as cytokine secretion through the senescence‐associated secretory phenotypes (SASPs), alterations in gene expression, and alternative splicing, as well as chromatin remodeling that includes senescence‐associated heterochromatin foci (SAHF) (Narita *et al*., 2003; Rodier *et al*., 2009; Kuilman *et al*., 2010; Campisi, 2013; Holly *et al*., 2013). Senescence is also seen as a mechanism that prevents tumorigenesis (Serrano *et al*., 1997). The upregulation of tumor suppressors, such as p16^INK4A^, p21, and p53, as well as the activation of RB, is common in senescent cells, contributing to irreversible cell‐cycle arrest (Beausejour, 2003; Sage *et al*., 2003; Kuilman *et al*., 2010; Campisi, 2013). Although replicative senescence is linked to telomere attrition (Allsopp *et al*., 1992; Bodnar *et al*., 1998), telomere shortening is not necessarily required for the onset of senescence, implying the existence of different senescent programs (van Deursen, 2014; Sharpless & Sherr, 2015). Consistent with this view, telomere‐independent senescence can be controlled by pathways triggered by insults (stress‐induced senescence), as well as by other intrinsic signals that occur during embryonic development and tissue repair (Von Zglinicki, 2002; Baker *et al*., 2008; Krizhanovsky *et al*., 2008; Schmidt *et al*., 2010; Nardella *et al*., 2011; Storer *et al*., 2013). Notably, senescence can also be engaged by the hyperactivation of factors, such as RAS, that promote cell growth, a process known as oncogene‐induced senescence that may be linked to telomere dysfunction (Courtois‐Cox *et al*., 2008; Günes & Rudolph, 2012). While the exact connection between senescence and organismal aging is still much debated (Sharpless & Sherr, 2015), it has become increasingly clear that cellular senescence plays a role in some age‐related diseases and in tissue degeneration associated with aging (Baker *et al*., 2008; Günes & Rudolph, 2012; van Deursen, 2014). Senescent cells progressively accumulate in the tissues and organs of aging mammals including humans (Herbig *et al*., 2006; Ressler *et al*., 2006; Jeyapalan *et al*., 2007; Kreiling *et al*., 2011). This accumulation of senescent cells may be due in part to a decreased ability of the immune system at removing them (Nikolich‐Zugich, 2008; Wang *et al*., 2011a). In addition, SASPs have been linked with aging organs and tissue degeneration (Parrinello *et al*., 2005; Coppé *et al*., 2008), where they are thought to enhance significantly the senescence of neighboring cells by a mechanism called paracrine senescence (Nelson *et al*., 2012; Acosta *et al*., 2013) (Fig. 1). Consistent with a role for senescence in aging, reducing the level of senescent cells is associated with a significant decrease in the incidence of age‐related disorders (Baker *et al*., 2008, 2011; Zhu *et al*., 2015), and was shown recently to improve homeostasis and extend lifespan in mouse models (Baker *et al*., 2016; Baar *et al*., 2017). While nonreplicating cells in aging tissues such as muscle harbor senescent markers and can become senescent, they may, in general, be more resistant to senescence. However, muscle replicative stem cells and satellite cells likely undergo senescence and may contribute more importantly to aging. Altogether, the accumulation of senescent cells in tissues and organs suggests that this process contributes to the progressive deterioration that irremediably associates with aging (van Deursen, 2014).

As senescence and aging are characterized by global cellular and molecular changes, it is fair to expect that splicing control will also be subjected to alterations. The challenge is to determine whether these changes are collateral or direct effects, and how they contribute to senescence and aging. Several reviews have recently presented splicing defects linked to age‐associated diseases, such as neurodegenerative disorders and cancer (Daguenet *et al*., 2015; Scotti & Swanson, 2015; Chabot & Shkreta, 2016). Here, our discussion will focus on recent progress achieved in documenting splicing alterations that directly contribute to cell senescence and aging, including accelerated aging (e.g., Hutchinson–Gilford progeria syndrome or HGPS).

## Basic concepts of alternative splicing

**Figure 2:**
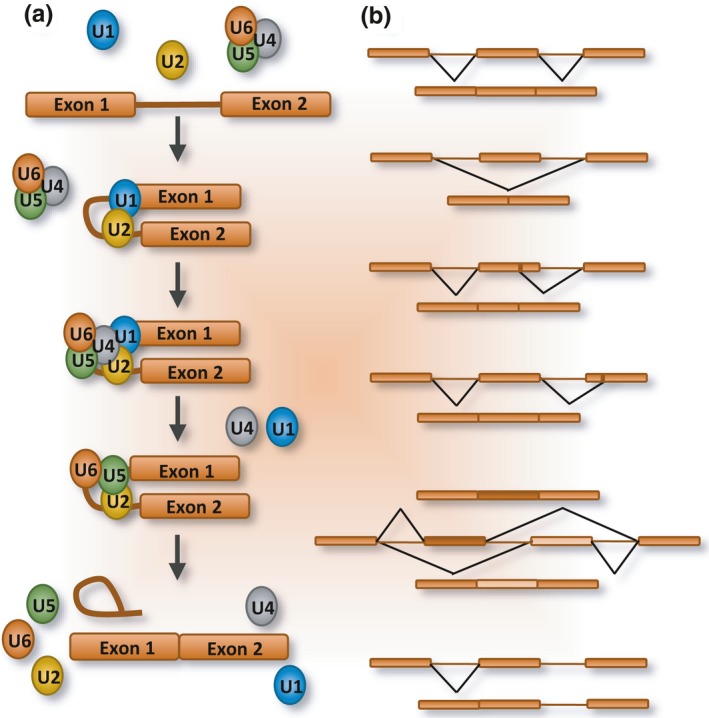
Constitutive and alternative splicing. (a) The U1 snRNP recognizes the 5′ splice site on the pre‐mRNA, while U2 snRNP interacts with the branchsite near the 3′ splice site. The U4/U5/U6 tri‐snRNP complex is then recruited. After the release of U1 and U4, the spliceosome first executes 5′ splice site cleavage coupled with branch formation. The second step (3′ splice site cleavage and exon ligation) then occurs, producing the mRNA and the excised intron. (b) Alternative splicing differentially combines exons or portions thereof to increase transcriptome diversity. In the case of exon skipping, the splice sites flanking the exon are not recognized leading it to be considered as part of an intron. Different modes of alternative splicing exist. From top to bottom: constitutive splicing, exon skipping, alternative 5′ splice site use, alternative 3′ splice site use, mutually exclusive exon inclusion, and intron retention.

The vast majority of precursor mRNAs (pre‐mRNAs) produced by mammalian cells are made of exons separated by introns. Introns are normally removed leaving joined exons that form the mature mRNA. Splicing is carried out by the spliceosome, a massive complex that includes hundreds of proteins and five small nuclear ribonucleoproteins (snRNPs) named U1, U2, U4, U5, and U6 (Matera & Wang, 2014). The U1 and U2 snRNPs, respectively, recognize the 5′ splice site and the branch site near the 3′ splice site, making these two snRNPs important in defining intron borders. While many introns are removed constitutively, a large fraction of splicing signals are not always used leading to alternative splicing (Fig. 2). Alternative splicing occurs in transcripts produced by more than 95% of human genes (Pan *et al*., 2008; Wang *et al*., 2008), including ones implicated in senescence, apoptosis, and DNA repair (Schwerk & Schulze‐Osthoff, 2005; Kelemen *et al*., 2013; Tang *et al*., 2013).

**Figure 3:**
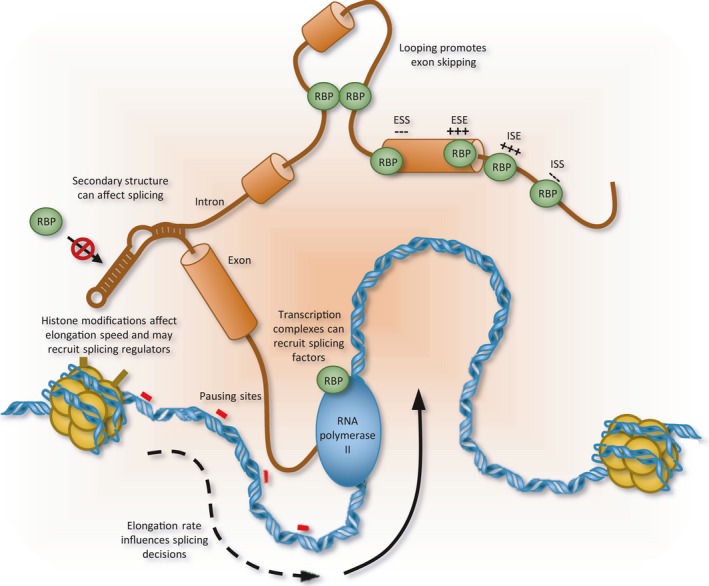
Control of alternative splicing. Exonic (ESS and ESE) and intronic (ISS and ISE) elements recruit RNA binding proteins (RBPs) to silence or enhance the use of splice sites. Interactions between RNA binding proteins may also reconfigure the architecture of the pre‐mRNA to affect splice site selection (Martinez‐Contreras *et al*., 2007). In addition, pre‐mRNA secondary structure may be inhibitory or may be used to approximate a regulatory element near a target splice site. Splice site selection can also be influenced by transcription. The speed of transcription will determine the time given for a complex to be assembled and influence selection when splice sites are in competition. In other cases, splicing regulators may be interacting with the polymerase complex or with chromatin to be deposited on the pre‐mRNA that emerges from the transcription complex. The presence of specific modifications on histones will impact the speed of transcription and the recruitment of adapters that in turn interact with splicing regulators.

Alternative splicing is a complex and tightly regulated process. Many RNA binding proteins act as splicing regulators to facilitate or inhibit splice site recognition by spliceosome components (Fu & Ares, 2014). Splicing regulators include, but are not limited to, members of the hnRNP and SR families of proteins (Busch & Hertel, 2012; Giulietti *et al*., 2013). The combinatorial cooperation or antagonism between distinct RNA binding proteins is thought to play a major role in conferring specificity to splicing decisions (Fu & Ares, 2014). Their interactions and their cellular localization can be modulated by post‐translational modifications (Howard & Sanford, 2015; Shkreta *et al*., 2016) that are often tightly integrated to environmental cues and homeostatic imbalances. Many splicing regulatory factors recognize short sequences within the pre‐mRNA, and these elements can be classified in four groups termed Intronic and Exonic Splicing Silencers (ISS and ESS, respectively) and Intronic and Exonic Splicing Enhancers (ISE and ESE, respectively). The availability of these elements and of the splice sites will vary depending on the secondary structure adopted by the pre‐mRNA (Jin *et al*., 2011). The position of these elements relative to splicing signals is often critical to determine whether they will have a positive or negative impact on splicing. For example, a RBFOX binding site positioned downstream of a 5′ splice site usually stimulates splicing, whereas the same site positioned in the upstream intron may promote exon skipping (Yeo *et al*., 2009) (Fig. 3).

In addition, the control of splice site selection is often coupled with transcription (Naftelberg *et al*., 2015). In this context, at least two mechanisms of control can exist. First, RNA polymerase II, transcription factors, and chromatin components can recruit generic and regulatory splicing factors. Second, change in the speed of transcription elongation and the presence of pausing sites linked to chromatin structure will affect the time given to splicing regulatory complexes to assemble and affect the use of a splice site in a competitive environment (Lee & Rio, 2015; Naftelberg *et al*., 2015; Nieto Moreno *et al*., 2015). One notable example of this coupling involves the DNA binding protein CTCF, which pauses the elongating polymerase to favor inclusion of exon 5 in the nascent *CD45* pre‐mRNA (Shukla *et al*., 2011). A developmentally induced DNA methylation event located in exon 5 inhibits the binding of CTCF to promote exon 5 skipping (Shukla *et al*., 2011).

It must also be pointed out that alternative splicing defects can arise when the levels of generic spliceosome components are altered. For example, the reduced expression of the protein SMN, that is implicated in snRNP assembly, leads to a wide‐range of alternative splicing alterations (Zhang *et al*., 2008). Likewise, mutations in the snRNP components PRPF8, PRPF3, U2AF35, and SF3B1 cause diseases (e.g., retinitis pigmentosa for PRPF proteins, and myelodysplasia for U2AF35 and SF3B1) that are associated with broad changes in the production of splice variants (Chabot & Shkreta, 2016). Splicing defects may occur because splice site selection and spliceosome kinetics are highly dependent on the concentration of core spliceosomal components, and that alternative splicing units often harbor weaker splice sites that may be more sensitive to drops in the levels of generic splicing factors.

## Aging and alternative splicing control

With such a complex regulatory machinery controlling splicing decisions, the molecular changes that occur with aging are therefore likely to impact the activity of factors that control splicing. A gene ontology analysis in both human and mouse reported that changes in pathways such as mRNA binding, RNA processing, and RNA splicing are strongly associated with age (Southworth *et al*., 2009; Harries *et al*., 2011). Age‐related splicing changes in the human brain affect pathways such as sugar metabolism and DNA repair (Tollervey *et al*., 2011), both relevant to aging (Colman *et al*., 2009; López‐Otín *et al*., 2013).

It is estimated that more than 50% of all age‐associated alterations in alternative splicing are due to changes in the expression of splicing factors (Mazin *et al*., 2013) (Table 1). Through microarray analysis, splicing changes have been linked to several splicing factors, including PTB (aka hnRNP I), ESRP1/ESRP2, NOVA1, and hnRNP K, whose expression also decreases with age (Tollervey *et al*., 2011). Recently, the expression of splicing regulators hnRNP A1 and A2 was linked positively to parental longevity in humans (Lee *et al*., 2016). Alterations in RBP expression may be caused by changes in the activity of transcription factors (Table 1). For instance, the activity of transcription factors such as STAT, IRF, GATA, and NF‐кB is presumed to vary with age (Stilling *et al*., 2014). Moreover, transcription factors can themselves be subject to age‐dependent alternative splicing that in turn may affect the expression of target genes. For example, microarray analysis showed that *STAT1* splicing was significantly disrupted in aging human peripheral blood cells (Harries *et al*., 2011), although it is unclear whether STAT1 splice variants display differences in their ability to activate the expression of target genes.

**Table 1:** Age‐related alteration of notable transcription (in bold) and splicing factors

Genes	Function	Age‐related alteration	Direction	Notes	References
***Ap1***	**Transcription**	**Expression of target genes**	**Up**	**Mice**	**Stilling** ***et al.*** **(** 2014 **)**
*Eftud2*	Splicing	Alternative splicing	N/A	Mice	Rodríguez *et al*. (2016)
*ESRP1*	Splicing	Expression	Up	Human	Tollervey *et al*. (2011)
*ESRP2*	Splicing	Expression	Up	Human	Tollervey *et al*. (2011)
***Ets***	**Transcription**	**Expression of target genes**	**Up**	**Mice**	**Stilling** ***et al.*** **(** 2014 **)**
***Gata***	**Transcription**	**Expression of target genes**	**Up**	**Mice**	**Stilling** ***et al.*** **(** 2014 **)**
*HnrnpH1*	Splicing	Alternative splicing	N/A	Mice	Stilling *et al*. (2014)
*HnrnpII*	Splicing	Alternative splicing	N/A	Mice	Stilling *et al*. (2014)
*HNRNPK*	Splicing	Expression	Down	Human	Tollervey *et al*. (2011)
*HrnrpA1*	Splicing	Alternative splicing	N/A	Mice	Stilling *et al*. (2014)
***Irf***	**Transcription**	**Expression, expression of target genes**	**Up**	**Mice**	**Stilling** ***et al.*** **(** 2014 **)**
***Nfkb1***	**Transcription**	**Expression of target genes**	**Up**	**Mice**	**Stilling** ***et al.*** **(** 2014 **)**
*NOVA1*	Splicing	Expression	Down	Human	Tollervey *et al*. (2011)
*Prpf3*	Splicing	Alternative splicing	N/A	Mice	Rodríguez *et al*. (2016)
*Prpf8*	Splicing	Alternative splicing	N/A	Mice	Rodríguez *et al*. (2016)
*PTBP1*	Splicing	Expression	Up	Human	Tollervey *et al*. (2011)
*PTBP2*	Splicing	Expression	Down	Human	Tollervey *et al*. (2011)
*RBFOX1*	Splicing	Expression	Down	Human	Tollervey *et al*. (2011)
*Sf3b1*	Splicing	Alternative splicing	N/A	Mice	Rodríguez *et al*. (2016)
*SLU7*	Splicing	Expression	Down	Human	Tollervey *et al*. (2011)
*SNRPB2*	Splicing	Expression	Down	Human	Tollervey *et al*. (2011)
***SpI***	**Transcription**	**Expression of target genes**	**Up**	**Mice**	**Stilling** ***et al.*** **(** 2014 **)**
*SRSF1*	Splicing	Alternative splicing	N/A	Human and mice	Stilling *et al*. (2014), Harries *et al*. (2011)
*Srsf11*	Splicing	Alternative splicing	N/A	Mice	Stilling *et al*. (2014)
*SRSF2*	Splicing	Expression	Down	Human	Tollervey *et al*. (2011)
*Srsf5*	Splicing	Alternative splicing	N/A	Mice	Stilling *et al*. (2014)
*RSF6*	Splicing	Alternative splicing	N/A	Human and mice	Stilling *et al*. (2014), Harries *et al*. (2011)
***STATs***	**Transcription**	**Alternative splicing, expression, expression of target genes**	**Up**	**Human and mice**	**Stilling** ***et al.*** **(** 2014 **), Harries** ***et al.*** **(** 2011 **)**

In the worm *Caenorhabditis elegans*, the enhanced longevity elicited by caloric restriction is compromised by depleting SFA‐1, which is the homolog of the mammalian splicing factor SF1, known to interact with the branchsite region (Heintz *et al*., 2016). Notably, overexpression of SFA‐1 is sufficient to increase worm longevity. The depletion of SFA‐1 preferentially promotes splicing defects in transcripts involved in metabolic processes including lipid catabolism and carbohydrate transport, and affects the activity of components of the TORC1 pathway (Heintz *et al*., 2016). Whether overexpression of SFA‐1 corrected the noted splicing defects has not been investigated. An important component of the TORC1 pathway is mTOR whose alternative splicing is controlled by Sam68 (Huot *et al*., 2012). Although overexpression of the splice variant mTORβ can promote tumorigenesis (Panasyuk *et al*., 2009), it is not known whether its production is affected during aging. However, ablation of Sam68 protects mice from age‐related loss of bone mass (Richard *et al*., 2005).

Age‐related splicing changes in mice can also be regulated in an unexpected manner. For instance, the age‐dependent nuclear translocation of the ion channel subunit protein P2X6 in mouse allows it to interact with and sequester the U2 snRNP protein SF3A1, decreasing overall splicing activity (Díaz‐Hernández *et al*., 2015). However, this phenomenon appears to saturate when mice reach adult age, suggesting that this process may be part of normal maturation and development rather than being associated with aging *per se*.

Most transcripts encoding splicing regulatory factors are themselves subject to alternative splicing. High‐throughput analyses revealed that the splicing profiles of *Hnrnph1, Hnrnpll, Hnrnpa1, Srsf5, Srsf6,* and *Srsf11* change through aging in mice, with human *SRSF6* and *SRSF1* being among the most altered by aging (Harries *et al*., 2011; Stilling *et al*., 2014). These changes in turn possibly alter the alternative splicing of numerous transcripts. Altogether, there is growing evidence documenting that the transcription and splicing of splicing regulators are altered in aging tissues. The accumulation of such alterations is likely to have a profound effect on the transcriptome of aging cells and the aging phenotype.

## Age‐related splicing alterations in growth regulators

Several genes produce transcripts whose alternative splicing changes in aging tissues or during prolonged cell passages in culture. An important question is whether those changes are the causes or the consequences of aging. Although recent studies have reported splicing changes in transcripts encoding proteins involved in processes that are intimately associated with aging, such as DNA damage sensing, DNA repair, and telomere biogenesis (Tollervey *et al*., 2011; Rodríguez *et al*., 2016), we currently do not know if these switches produce splice variants with distinct functional properties. Here we will restrict our discussion to splice variants for which experimental evidence support a role in aging or senescence.

### p53

One of the best known and possibly most important growth regulators is the tumor suppressor protein p53 that is implicated in cell‐cycle arrest, senescence, and apoptosis (Vogelstein *et al*., 2000; Lowe *et al*., 2004). Mice lacking p53 develop normally but are highly predisposed to spontaneous tumor formation (Donehower *et al*., 1992). Most human cancers have a mutated p53 or express low level of wild‐type p53 (Kamijo *et al*., 1998; Onel & Cordon‐Cardo, 2004). The role of p53 in human aging is supported by epidemiological studies (van Heemst *et al*., 2005; Ørsted *et al*., 2007), and the fact that p53 regulates several processes relevant to organismal aging (Rufini *et al*., 2013); p53 can promote apoptosis, the DNA damage response, autophagy, and mitophagy (Eisenberg *et al*., 2009; Liang, 2010; Gao *et al*., 2011; Wang *et al*., 2011b; Tucci, 2012). Although p53 contributes to senescence‐associated replicative arrest, it can restrain SASP (Coppé *et al*., 2008; Raj & Attardi, 2013).

**Figure 4:**
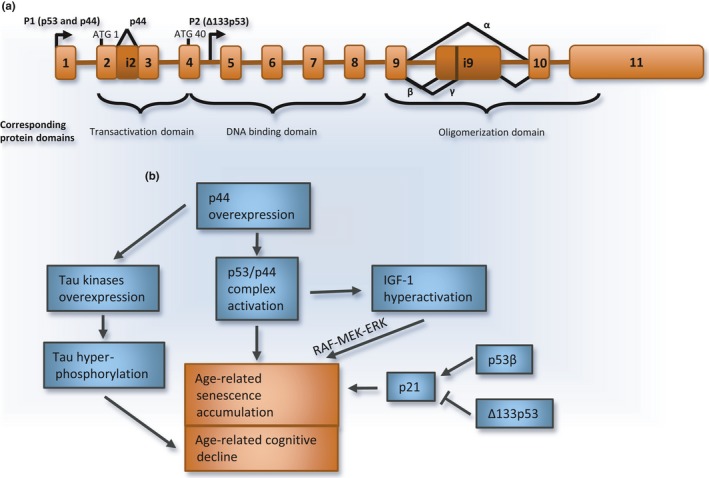
p53 splice variants and aging. (a) p53 alternative splicing and the production of splice variants. The structure of the *p53* gene is illustrated with exons and introns, alternative start sites of transcription (P1 and P2 arrows), and translation initiation codons (ATG). p44 is produced when intron i2 is retained, eliciting the use of a downstream initiation codon in exon 4. The use of the alternative start site (P2) leads to the production of the Δ133p53 isoform. Complex alternative splicing can occur within intron 9 to produce β or γ (α is indicated here as the simplest case where the whole intron i9 is removed). Both p44 and Δ133p53 lack the transactivation domain. (b) p44 forms a complex with p53 to regulate its activity. When overexpressed, p44 leads to senescence and hyperactivates the IGF‐1 pathway that in turn promotes cell‐cycle arrest through the RAF‐MEK‐ERK pathway. p44 is also linked to age‐related cognitive decline as its overexpression upregulates tau kinases. Variants p53β and Δ133p53 also regulate senescence by, respectively, activating and inhibiting the cell‐cycle arresting protein p21.

The alternative splicing of *p53* transcripts generates the truncated variant p44 that lacks the transactivation domain (Fig. 4a), and whose production increases in aging mice (Pehar *et al*., 2014). Notably, p44 can also be produced by an internal ribosome entry site (IRES) in the p53 mRNA. This mechanism occurs preferentially at the G1‐S cell‐cycle transition and is not inducible by stress (Courtois *et al*., 2002; Ray *et al*., 2006). The respective contribution of the splicing‐mediated and the IRES‐mediated processes to the production of p44 in aging cells is unclear. What is clear however is that overexpression of p44 in transgenic mice (p44^+/+^) leads to an accelerated age‐associated phenotype, suppresses cell proliferation, and increases senescence (Maier *et al*., 2004) (Fig. 4b). These effects do not occur in p44 mice lacking p53, indicating that full‐length p53 is necessary to elicit the aging phenotype (Maier *et al*., 2004). Notably, p47 (the human orthologue of p44) interacts with full‐length p53 to modulate p53 activity (Courtois *et al*., 2002; Ghosh *et al*., 2004). Compared to the p53 homodimer, the p44/p53 complex displays distinct half‐life, post‐translational modifications, and promoter affinity (Campisi, 2004; Scrable *et al*., 2005). Interestingly, the accelerated aging and growth arrest caused by disrupting the balance between p53 and p44 is functionally linked to the pathway initiated by the Insulin‐like Growth Factor‐1 (IGF‐1) (Maier *et al*., 2004), which extends lifespan in *C. elegans, D. melanogaster,* and mice when inhibited. p53 modulates the IGF‐1 pathway by decreasing the expression of the IGF‐1 receptor (IGF‐1R) (Werner *et al*., 1996). In contrast, mice overexpressing p44 display high levels of IGF‐1/IGF‐1R (Pehar *et al*., 2010). Notably, the oncogenic MEK protein kinase has been associated with senescence when it is hyperactivated in a mouse model (Lin *et al*., 1998). Hyperactivation of the RAF‐MEK‐ERK pathway by high levels of IGF‐1 induces cell‐cycle arrest (Maier *et al*., 2004). This effect is considered a fail‐safe mechanism that increases senescence, and it may explain at least in part the aging defect of p44^+/+^ mice (Campisi, 2004; Maier *et al*., 2004). p44^+/+^ transgenic mice also display premature synaptic deficit, cognitive decline as well as Alzheimer's disease‐like features, possibly associated with the abnormal phosphorylation of the microtubule‐binding protein tau (Pehar *et al*., 2010). In fact, p44 is reported to bind, independently of p53, to the promoter of several tau kinases genes resulting in their overexpression (Pehar *et al*., 2014). Thus, altering the balance between p53 variants can affect aging in mice (Campisi, 2004; Maier *et al*., 2004; Pehar *et al*., 2014), although it is unclear if the above conclusions apply to human aging.

In addition to p44, other p53 variants participate in the onset of senescence. In contrast to p53, whose level remained unchanged, the ∆133p53 and p53β splice variants (Fig. 4a) are, respectively, downregulated and upregulated in human senescent fibroblasts (Fujita *et al*., 2009). This functional link is supported by the fact that the knockdown of ∆133p53 induces robust senescent phenotypes, such as flattened morphology, an increase in β‐galactosidase activity, and p21 overexpression. In contrast, upregulation of ∆133p53 induces cell proliferation, as well as repression of p21 and the E3 ubiquitin ligase MDM2 involved in p53 degradation; while expression of p53β has the opposite effects (Fujita *et al*., 2009). Overexpressing either ∆133p53 or p53β in p53‐null fibroblasts had no effect, indicating that these variants need to interact with full‐length p53 to impact the senescent program (Fujita *et al*., 2009).

Despite clear differences in the function of p53 splice variants, little is known about what regulates their production by alternative splicing. The SR protein SRSF3 interacts with the *p53* pre‐mRNA (Tang *et al*., 2013) and downregulates the production of p53β (Tang *et al*., 2013). Moreover, senescent human fibroblasts and p53β overexpressing cells display reduced levels of SRSF3 mRNA. A drop in SRSF3 is not seen in oncogenic RAS‐induced senescence, suggesting that the role of SRSF3 is specific to replicative senescence. The knockdown of SRSF3 also induces a senescent phenotype, p53 phosphorylation and an increase in p53β (Tang *et al*., 2013). These findings suggest a crucial role for SRSF3 in preventing senescence, but additional analyses in tissues are needed to confirm whether this observation is relevant to normal aging.

### IGF‐1

While IGF‐1 is expressed in nearly every tissue, it is mostly produced by the liver, from which it enters the general circulation (Flier *et al*., 1997; Sjögren *et al*., 1999). IGF‐1 is an important player in normal growth and development. Mutations that reduce IGF‐1 signaling increase the lifespan of *D. melanogaster* and *C. elegans* (Altintas *et al*., 2016). Caloric restriction, a condition that increases longevity in several organisms, promotes a drop in IGF‐1 synthesis and signaling (Sjögren *et al*., 1999; Campisi, 2004; Tucci, 2012; Higashi *et al*., 2014). The *IGF‐1* transcript undergoes alternative splicing to produce at least three mRNA variants: IGF‐1Ea is the most abundant variant lacking exon 5; IGF‐1Eb is only found in humans and lacks exon 6; and IGF‐1Ec, also known as the Mechano Growth Factor (MGF), contains both exons 5 and 6, and is associated with satellite cell activation, proliferation, and skeletal muscle repair (Yang & Goldspink, 2002; Goldspink, 2005). Exon 5 of *IGF‐1* contains a regulatory ESE that recruits SRSF1 to promote exon 5 inclusion (Smith *et al*., 2002).

The splicing variants IGF‐1Ea and MGF affect different pathways. IGF‐1Ea through its receptor IGF‐1R activates both the RAF‐MEK‐ERK and the PI3K‐AKT pathways that are respectively associated with proliferation and differentiation (Coolican *et al*., 1997; Stavropoulou *et al*., 2009). MGF activates in an IGF‐1R‐independent manner the RAF‐MEK‐ERK pathway only, supporting its role in proliferation and skeletal muscle repair (Philippou *et al*., 2009; Stavropoulou *et al*., 2009).

Early studies on *IGF‐1* splice variants revealed that their relative production did not significantly change with age. However, in contrast to IGF‐1Ea, MGF levels are significantly induced after exercise in young compared to older rats, as well as in humans (Owino *et al*., 2001; Hameed *et al*., 2003). Based on these results, it was suggested that the age‐associated decline in skeletal muscle repair and maintenance (Brooks & Faulkner, 1990) may be related to a progressive loss of MGF production after exercise.

### SIRT1

The sirtuin SIRT1 is the mammalian orthologue of yeast sir2 and is an NAD^+^‐dependent histone deacetylase (HDAC). SIRT1 has important functions in gametogenesis, embryonic development, skeletal muscle differentiation, and homeostasis (Cheng *et al*., 2003; Fulco *et al*., 2003; McBurney *et al*., 2003; Feige & Johan, 2008). SIRT1 also regulates inflammation by interfering with the NF‐кB pathway (Salminen *et al*., 2008; Shinozaki *et al*., 2014) and represses p53 activity to reduce apoptosis during stress (Luo *et al*., 2001; Cheng *et al*., 2003).

Sir2/SIRT1 activation extends lifespan in yeast, *C. elegans*,* Drosophila*, as well as in mice (Kaeberlein *et al*., 1999; Tissenbaum & Guarente, 2001; Whitaker *et al*., 2013; Mitchell *et al*., 2014). Although sirtuins are considered anti‐aging proteins (Guarente, 2007), the role of SIRT1 in human aging remains controversial (Ledford, 2011; Lombard *et al*., 2011). For instance, while resveratrol stimulates the interaction between SIRT1 and lamin A, whose importance in aging defects is well documented (Liu *et al*., 2012), activation of SIRT1 by resveratrol has no demonstrated impact on health or longevity (Semba *et al*., 2014). In mammals, the activation of SIRT1 reduces the level of circulating IGF‐1, also consistent with the caloric restriction model (Brown *et al*., 2010). Sir2 can activate autophagy (Morselli *et al*., 2010) and may enhance longevity by producing more homogeneously healthy mitochondria (Nemoto *et al*., 2005). It has been proposed that the drop in NAD^+^ level that occurs in aging tissues (Braidy *et al*., 2011; Massudi *et al*., 2012) decreases the activity of SIRT1 leading to a progressive accumulation of reactive oxygen species (ROS) and mitochondrial dysfunction (López‐Otín *et al*., 2013; Kim *et al*., 2014).

*SIRT1* undergoes alternative splicing of exon 8 to produce SIRT1∆8 which lacks a portion of the deacetylase domain. SIRT1∆8 expression rises after stresses, and while this variant by itself displays reduced p53 deacetylation activity, it exerts an additive deacetylation effect on p53 when expressed with full‐length SIRT1 (Lynch *et al*., 2010). Combining stresses with the depletion of p53 also leads to an overexpression of *SIRT1∆8* mRNA and protein, while activating p53 has the opposite effect (Lynch *et al*., 2010). In stress conditions, the depletion of SIRT1∆8 leads to an abnormal accumulation of p53, accompanied by a significant increase in apoptosis, consistent with a repressive role for SIRT1Δ8 in p53 regulation after stress (Lynch *et al*., 2010).

**Figure 5:**
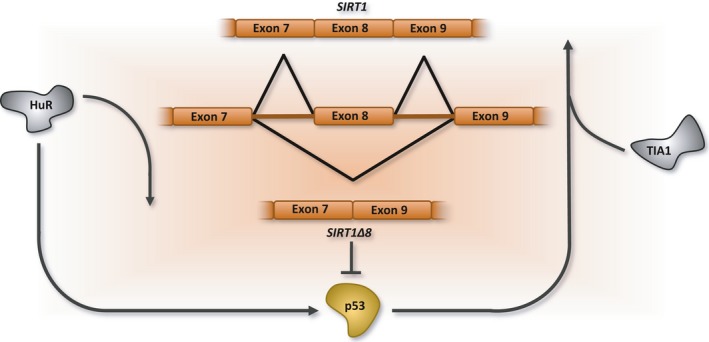
Regulating the alternative splicing of *SIRT1*. Skipping of exon 8 in the *SIRT1* pre‐mRNA generates SIRT1Δ8. The HuR splicing factor enforces the production of SIRT1Δ8, while TIA1 and p53 (by an unknown mechanism) show the opposite effect. However, the upregulation of SIRT1Δ8 inhibits p53 leading to reciprocal regulation and a negative feedback loop.

A stress‐induced drop in the expression of SRSF2 has been implicated in the production of the SIRT1∆8 variant (Lynch *et al*., 2010). *SIRT1* splicing implicates RNA binding proteins that are also linked to p53 activity. Overexpression of RNA binding proteins TIA1 or TIAL1 promotes *SIRT1* exon 8 inclusion and inhibits cell growth (Zhao *et al*., 2014), while their knockdown has the opposite effect. A gene ontology analysis reveals that the tumor suppressor phenotype triggered by TIA1/TIAL1 overexpression involves the upregulation of p53 targets (Sánchez‐Jiménez *et al*., 2015). Conversely, the RNA binding protein HuR, which enhances p53 mRNA translation by binding to its 3′ UTR (Mazan‐Mamczarz *et al*., 2003; Abdelmohsen *et al*., 2014), also promotes *SIRT1* exon 8 skipping when overexpressed, while the opposite effect is observed following HuR knockdown (Zhao *et al*., 2014). Taken together, the p53‐dependent alternative splicing of *SIRT1* exon 8 and the reciprocal regulatory relationship with p53 suggest a negative feedback loop (Lynch *et al*., 2010) (Fig. 5) that may be important to prevent abnormal cell growth. Notably, both suboptimal and excess levels of SIRT1 have a tumor suppressive impact in rodents (Firestein *et al*., 2008; Kabra *et al*., 2009). Thus, while SIRT1 may play an important role in preventing tumor formation, the role of SIRT1 and its splice variant in senescence and apoptosis remains to be better understood.

**Figure 6:**
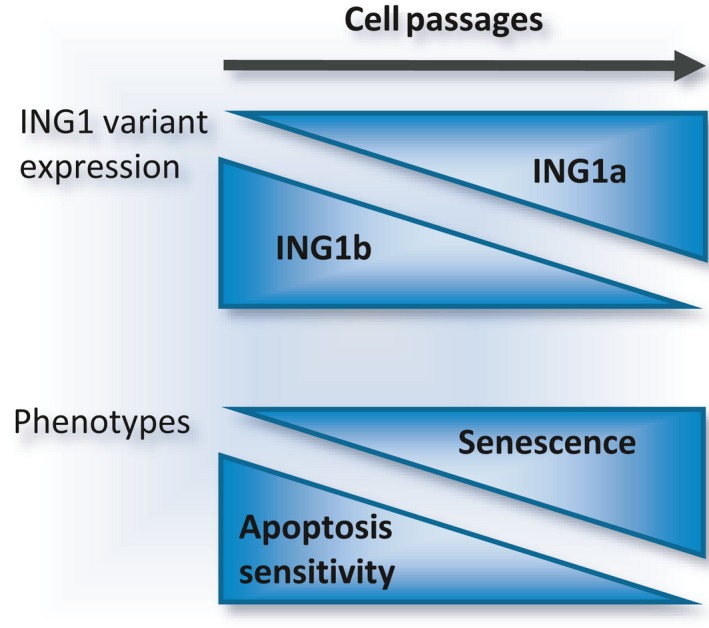
ING1 splice variants and senescence. *ING1* pre‐mRNA alternative splicing generates two variants termed ING1b and ING1a. In young cultured cells, ING1b is predominant and, when overexpressed, can trigger apoptosis through p53 acetylation. ING1a production increases when cells approach senescence in culture. ING1a elicits several senescence phenotypes when ectopically overexpressed.

### ING1

ING1 functions in apoptosis, senescence, and DNA repair (Kichina *et al*., 2006; Coles *et al*., 2007; Peña *et al*., 2008; Abad *et al*., 2011). The expression of ING1 is ubiquitous, p53‐independent and is frequently reduced in human cancers, supporting its role as a tumor suppressor (Toyama *et al*., 1999; Cheung *et al*., 2000). Consistently, knocking out *ING1* provokes a high frequency of spontaneous tumor development in mice (Kichina *et al*., 2006). *ING1* produces at least four mRNA isoforms of which two are splice variants: *ING1a* and *ING1b* (Feng *et al*., 2002). INGb, but not ING1a, interact with HDAC complexes, and only overexpression of ING1b leads to increased acetylation of histones H3 and H4 (Skowyra *et al*., 2001; Vieyra *et al*., 2002a).

The level of ING1a increases at the expense of ING1b when cells approach senescence, suggesting a role for ING1a in cell‐cycle arrest (Soliman *et al*., 2008). Consistent with this view, cells overexpressing ING1a display senescence phenotypes such as formation of SAHF, flat morphology, and expression of senescence‐associated β‐galactosidase and p16 (Soliman *et al*., 2008). While ING1b also participates in senescence through upregulation of p16 (Li *et al*., 2011), it sensitizes young cells to apoptosis (Scott *et al*., 2001; Vieyra *et al*., 2002b; Guérillon *et al*., 2013) (Fig. 6). ING1b‐mediated apoptosis may occur by stabilization of p53, possibly by inhibiting deacetylation mediated by SIRT1 (Vaziri *et al*., 2001; Kataoka *et al*., 2003; Binda *et al*., 2008; Guérillon *et al*., 2013). The splicing shift toward ING1a may help to maintain the senescence status and thus may prevent apoptosis. Overall, the fact that splice variants of *p53* or *ING1* can differentially trigger senescence *in vitro* or age‐related phenotypes *in vivo* suggests that their alternative splicing control is directly implicated in aging.

## Alternative splicing and age‐related diseases

A number of human diseases known as progeroid syndromes provoke phenotypic alterations that resemble those noted during normal aging. These include Hutchinson–Gilford Progeria syndrome (HGPS or progeria), Werner syndrome, and Cockayne syndrome. The study of HGPS has revealed that alternative splicing defects are responsible for some of its distinctive features.

### Progeria

**Figure 7:**
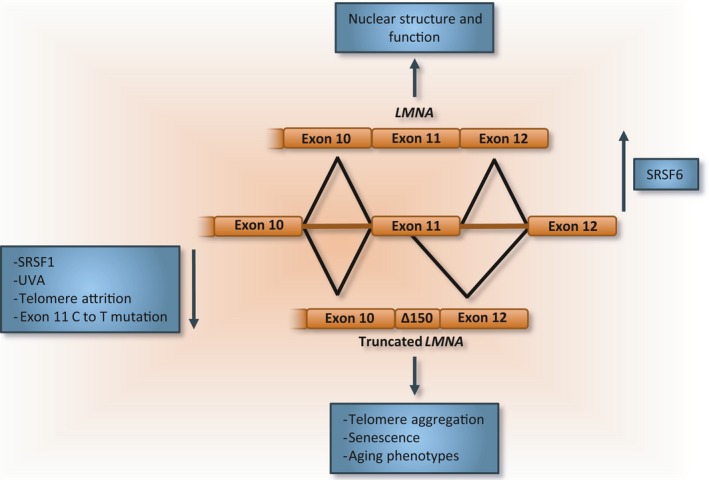
The alternative splicing of *LMNA* produces the progerin variant that is associated with aging and age‐related diseases. The use of an alternative 5′ splice site in exon 11 of the *LMNA* pre‐mRNA produces a truncated mRNA variant encoding progerin. In the primary form of progeria, a silent C to T mutation in exon 11 increases the use of this cryptic site by 50‐fold. Ectopic expression of progerin causes senescence, telomere aggregation, and a progeroid phenotype in animal models. The expression of progerin can also be triggered by UVA, telomere attrition, overexpression of SRSF1, or the depletion of SRSF6.

Progeria or HGPS is defined by a premature aging phenotype associated with a high incidence of age‐related disorders, such as cardiovascular impairment and atherosclerosis (Merideth *et al*., 2008; Hisama *et al*., 2011). Premature cell senescence has also been reported, consistent with the notion that senescence is associated with aging (Huang *et al*., 2005). While the relationship between HGPS and normal physiological aging is still debated (Burtner & Kennedy, 2010), tissues from HGPS patients are considered a good model for research on vascular aging as HGPS shares common mechanisms with age‐related vascular dysfunction (Brassard *et al*., 2016). Lamin proteins are components of the nuclear lamina and play key roles in nuclear structure and in processes such as transcription and DNA replication (Goldman *et al*., 2002; Dechat *et al*., 2008, 2009). Lamin A interacts with and activates SIRT1 (Liu *et al*., 2012). The *LMNA* gene normally encodes both lamin A and C proteins. A silent C to T mutation in exon 11 has been implicated in the most common form of HGPS (Hutchison, 2002; Eriksson *et al*., 2003). The mutation increases by 50‐fold the use of an alternative 5′ splice site situated 5 nucleotides upstream of the mutation, producing a variant lacking 150 nucleotides and a truncated protein called progerin lacking the corresponding 50 amino acids (Fig. 7). Progerin is abnormally processed and becomes sequestered in the nuclear membrane, decreasing the acetylase activity of SIRT1. Notably, progerin is detected in trace amounts in healthy tissues that lack the mutation. This observation and the fact that progerin is also produced in other animals (Lopez‐Mejia *et al*., 2011) suggest that progerin may have a normal physiological function (Hutchison, 2002; De Sandre‐Giovannoli *et al*., 2003; Eriksson *et al*., 2003; Scaffidi & Misteli, 2006; Liu *et al*., 2012). Lamins, including progerin, may regulate senescence pathways in patients with HGPS and healthy individuals (Karlseder, 2002; Freund *et al*., 2012; Wood *et al*., 2014). Notably, physical insults like UVA, but not UVB, drastically change the splicing profile of normal *LMNA* to favor the production of progerin in cultured human fibroblasts (Ikehata & Ono, 2011; Takeuchi & Rünger, 2013). In contrast to UVB, which preferentially produce pyrimidine dimers, the indirect generation of oxidative DNA damage through ROS induction by UVA (Cadet *et al*., 2009) may explain the upregulation of progerin. Thus, the UVA‐induced increase in progerin production may accelerate skin aging (Takeuchi & Rünger, 2013). It has also been noted that the production of progerin in an HGPS mouse model associates with impaired developmental splicing and an altered production of splice variants in the skin (Rodríguez *et al*., 2016).

Although the mRNA level of progerin does not significantly increase in aging biopsies (Scaffidi & Misteli, 2006; Rodriguez *et al*., 2009; Cao *et al*., 2011), the accumulation of progerin in the nuclear membrane, as reported in HGPS and healthy tissues, is believed to contribute to senescence. Consistent with this view, ectopic expression of progerin can induce progeroid phenotypes, such as nuclear misshape, premature senescence (Candelario *et al*., 2008), and telomere aggregation and dysfunction independently of telomere attrition (Benson *et al*., 2010; Cao *et al*., 2011). Also, the depletion of progerin downregulates expression of *p21*, as well as that of growth regulators IGFBP3 and GADD45B (Varela *et al*., 2005; Scaffidi & Misteli, 2006). Moreover, shortened telomeres induce the production of progerin, suggesting a positive feedback loop that ultimately converges into activation of senescence in normal tissues (Cao *et al*., 2011).

The above results suggest that the abnormal accumulation of progerin contributes to senescence and accelerated aging. As this accumulation also occurs during the normal aging process, the same senescence pathway may be activated in healthy individuals (Scaffidi & Misteli, 2006; McClintock *et al*., 2007). Despite several aging phenotypes displayed by patients with HGPS, an increase in the incidence of cancer is not one of them (Hennekam, 2006; Coppedè & Migliore, 2010). Based on these observations, it is tempting to suggest that progerin, while protecting the organism from tumorigenesis, accelerates the accumulation of senescence leading to degenerative phenotypes. Notably, patients with HGPS also do not show any increase in the incidence of Alzheimer's disease, potentially providing insight into protective mechanisms (Nelson *et al*., 2011).

The splicing regulatory proteins SRSF1 and SRSF6 bind close to the progerin‐producing splice site and elicit mutually antagonistic effects in both HeLa cells and primary HGPS fibroblasts; SRSF6 decreases the use of the progerin‐specific 5′ splice site, while SRSF1 enhances it (Lopez‐Mejia *et al*., 2011). Consistent with this result, the depletion of SRSF6 increases progerin accumulation in the nucleus of HGPS fibroblasts, while depletion of SRSF1 reduces the level of progerin and nuclear misshape (Lopez‐Mejia *et al*., 2011). Notably, the C to T mutation in exon 11 of *LMNA* increases the accessibility of the progerin 5′ splice site, which is normally sequestered in a conserved duplex structure (Lopez‐Mejia *et al*., 2011).

### Vascular aging

Vascular aging is characterized by a decline of endothelial function leading to a progressive deficiency in cardiovascular repair mechanisms (Ribera‐Casado, 1999) and vascular walls remodeling (Brandes *et al*., 2005; Minamino & Komuro, 2007). The decreasing proliferative potential of endothelial cells and the accumulation of senescent cells also deleteriously affect angiogenesis, nutrient trafficking, homeostasis, and the response to TGF‐β (Dimri *et al*., 1995; Foreman & Tang, 2003). Alternative splicing plays a crucial role in vascular integrity, and defective splicing leads to cardiovascular diseases such as aortic aneurysm and arrhythmias (Martin *et al*., 2014; Dlamini *et al*., 2015; Rizzacasa *et al*., 2017). For instance, the proportion of titin splice variants, which contribute to cardiac myocytes stiffness, is altered in heart diseases (LeWinter & Granzier, 2013). Alternative splicing of the titin transcript is controlled by RBM20, which acts on other pre‐mRNAs whose splicing is linked to dilated cardiomyopathy and other vascular diseases (Guo *et al*., 2012; Li *et al*., 2013; Maatz *et al*., 2014). Splicing regulators like hnRNP A1 and PTBP1 also contribute to cardiovascular risk factors including the metabolism of cholesterol, omega‐3, and omega‐6 (Medina *et al*., 2011; Mozaffarian & Wu, 2011; Reardon *et al*., 2011; Yu *et al*., 2014). Given that aging is a risk factor to vascular diseases, it is likely that age‐dependent alterations in at least some of the above splicing events and regulators contribute to cardiovascular diseases.

One example of age‐related splicing change in the vascular context concerns the *ENG* gene which codes for the glycoprotein endoglin, a transmembrane co‐receptor of TGF‐β that functions in endothelial cell growth, angiogenesis, differentiation, and senescence. The TGF‐β receptor complex includes endoglin and the TGF‐β type I and II receptors. The type I receptor associates with two antagonistic proteins, ALK1 and ALK5, that regulate the signal induced by TGF‐β (Bernabeu *et al*., 2007; ten Dijke & Arthur, 2007). The *ENG* pre‐mRNA is alternatively spliced to produce at least two mRNA variants: the long (L) and the short (S) version. The S variant has a retained intron that provides a premature stop codon before the last exon (Bellón *et al*., 1993; Pérez‐Gómez *et al*., 2005). While L‐endoglin is predominantly produced in young vascular endothelial cells, a shift toward the S form occurs during normal aging, as well as at in late passages in cell culture (Blanco *et al*., 2008; Blanco & Bernabeu, 2011). S‐endoglin is more likely to interact with ALK5 than ALK1, whereas L‐endoglin has more affinity for ALK1. Thus, the cellular responses mediated by TGF‐β signaling may depend on the alternative splicing of endoglin as the levels of ALK1 and ALK5 do not significantly change during senescence (ten Dijke & Arthur, 2007). L‐endoglin has a proliferative and pro‐angiogenic effect on endothelial cells through its interaction with ALK1, while S‐endoglin is more likely to induce a senescence phenotype (Lebrin *et al*., 2004; Düwel *et al*., 2006). Endothelial cells of transgenic mice overexpressing S‐endoglin show a lower proliferative rate, suggesting that S‐endoglin may contribute to senescence and age‐associated vascular pathologies (Blanco *et al*., 2008).

SRSF1 has been implicated in regulating the production of S‐ and L‐endoglins. Overexpressing SRSF1 increases S‐endoglin mRNA, possibly by interfering with branch site usage to promote intron 2 retention in *ENG* transcripts (Blanco & Bernabeu, 2011).

### Alzheimer's disease (AD)

Among age‐related disorders, AD is characterized by progressive neurodegenerative phenotypes leading to memory loss and dementia (Burns & Iliffe, 2009), possibly caused by aggregation of amyloid beta (Aβ) in the brain (Zheng & Koo, 2011; Kandalepas & Vassar, 2014). Aβ is the product of two subsequent enzymatic digestions driven, respectively, by the β‐secretase (BACE) and the γ‐secretase; the initial substrate for these enzymes is the amyloid precursor protein (APP) (Citron *et al*., 1995; Koffie *et al*., 2011; Zheng & Koo, 2011; Chami & Checler, 2012; Kandalepas & Vassar, 2014). BACE turns out to be a more desirable target against AD as γ‐secretase is believed to be more critical for normal function (Mowrer & Wolfe, 2008). The complex alternative splicing profile of BACE exons 3 and 4 generates at least four splice variants: I‐501, I‐476, I‐457, and I‐432 (Mowrer & Wolfe, 2008; Fisette *et al*., 2012). While total level of *BACE* transcripts remains unchanged when comparing young and old mice, the level of full‐length I‐501 increases in an age‐dependent manner (Zohar *et al*., 2005). Considering the strong correlation between BACE activity and Aβ accumulation in the brain (Hsiao *et al*., 1996; Tanahashi & Tabira, 2001), a rise in BACE level and the consequent accumulation of Aβ may increase the predisposition to AD as well as to other age‐associated cognitive impairments.

In addition, age‐related expression and splicing changes have been noted both in *BACE* and *APP* in the mice hippocampus (Stilling *et al*., 2014). Interestingly, overexpression of APP in AD mice model alters *BACE* alternative splicing in the brain, suggesting a feedback role for APP on Aβ production through *BACE* splicing (Zohar *et al*., 2005).

Little is known about the control of alternative splicing of *BACE1* transcripts. An ESE potentially recognized by SRSF2 positively regulates the inclusion of exon 4 (Mowrer & Wolfe, 2009). The exon 3 and exon 4 contain several alternative splice sites leading to a complex splicing profile whose regulation is not understood. Notably, a G‐rich sequence within exon 3 controls 5′ splice site selection to enhance production of the full‐length mRNA. This G‐rich sequence forms a G‐quadruplex structure that interacts with the splicing factor hnRNP H (Fisette *et al*., 2012). Decreasing the level of hnRNP H by RNA interference significantly reduces production of full‐length *BACE,* hence decreasing Aβ production (Zheng & Koo, 2011; Chami & Checler, 2012; Fisette *et al*., 2012). These findings identify potential targets for therapies that may help reduce the damaging effects of the Aβ accumulation in the brain observed in AD and normal aging, and show the essential role that alternative splicing and its regulation play in such diseases. It is worth restating that patients with HGPS are largely spared from Alzheimer's disease and that they may represent a unique cohort to study the underlying protective mechanisms.

## Conclusions and perspectives

**Figure 8:**
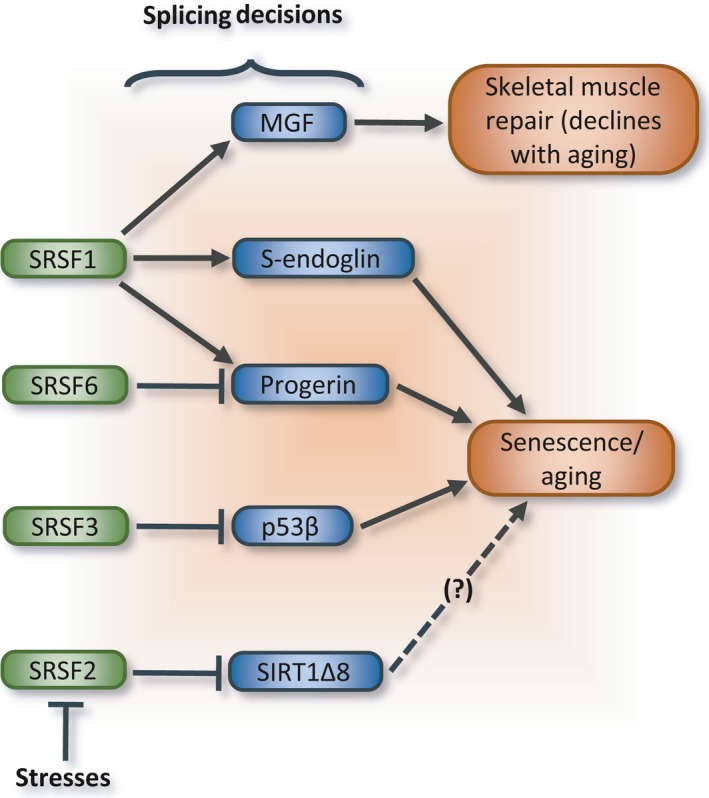
Contribution of SR proteins to senescence and the aging phenotype. As indicated, SRSF1, SRSF2, SRSF3, and SRSF6 enhance or repress the production of specific splice variants implicated in age‐related phenotype, aging, and senescence.

Given the challenges associated with maintaining homeostasis in cells and tissues subjected to constant internal and external insults, we can anticipate that a subset of mutations and epigenetic changes may alter the expression or activity of spliceosome components and splicing regulatory factors. These changes may, in turn, alter the splicing profile in several transcripts, resulting in a cascade of alterations that may either activate senescence, promote apoptosis, or elicit tumor formation. Although senescence and apoptosis may protect against tumor formation, the gradual accumulation of senescent cells will elicit tissue degeneration and organ dysfunction. While progressive age‐related disturbances in homeostasis do indeed correlate with a broad range of alterations in alternative splicing, the current challenge is to determine whether a specific splicing change contributes to the aging phenotype or is simply a consequence with little or no functional impact. In this review, we have focused on altered alternative splicing events whose contributions to age‐related phenotypes are experimentally supported. These events occur in genes known for their implication in mechanisms that are relevant to cell senescence and organismal aging. Although we reviewed the impact of selected splice variants on aging, regulatory networks likely coordinate the production of splice variants from different genes to maximize functional outcomes that determine cell fate, and ultimately the aging phenotype. Consistent with this proposition, the activity of p53 in senescence and apoptosis can be modulated by SIRT1 and ING1, in turn affecting ING1 signaling and SIRT1 activity. Extending these relationships to the full repertoire of splice variants for all the components of the extended p53 regulatory network may be required to determine how important is the level of coordination and feedback involved in the production of splice variants contributing to aging. Already, the splicing regulatory proteins SRSF1, SRSF2, SRSF3, and SRSF6 are emerging as central players coordinating multiple splicing decisions in age‐relevant and senescent transcripts (Fig. 8). Future studies will likely investigate in more details how defects in the expression or activity of these proteins, as well as hnRNP proteins and core spliceosomal components, affect senescence and aging. Another group of potentially relevant regulatory molecules are noncoding RNAs. Some display age‐dependent changes in expression (e.g., see Gruner *et al*., 2016), and recent studies have implicated microRNAs, large noncoding RNAs, and circular RNAs in senescence and SASP (Abdelmohsen & Gorospe, 2015; Panda *et al*., 2017a). Specifically, the circular RNA CircPVT1, whose expression is reduced in senescent cells, is produced by circularization of an exon of the *PVT* gene through RNA splicing. CircPVT sequesters *let‐7* RNA and its depletion triggers senescence (Panda *et al*., 2017b). There is clearly a need to investigate further how splicing regulation is altered to affect the production of circular RNA molecules during senescence and aging. To help clarify the contribution of an expanding list of splice variants and regulators associated with aging, it would be useful to combine expression assays with the monitoring of phenotypes like cell growth and the production of senescent markers. Likewise, it would be informative to determine whether and how SASP components produced by senescent cells reprogram the splicing profiles of neighboring cells.

**Figure 9:**
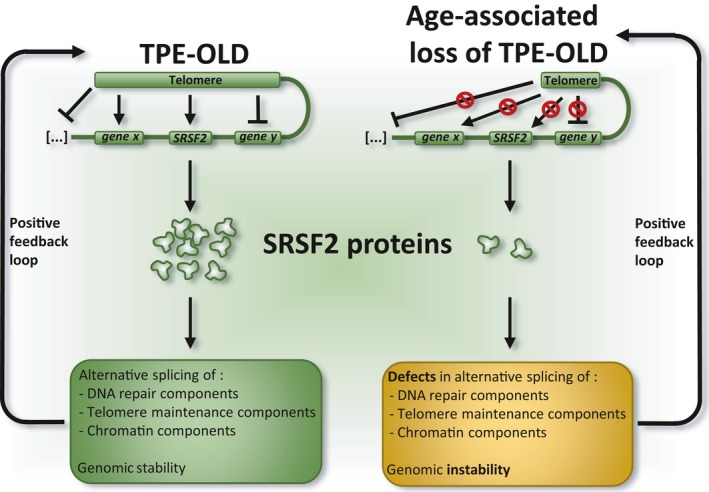
A model that links telomere function with splicing control. Telomere erosion would abrogate TPE‐OLD to alter the expression/activity of splicing regulators such as SRSF2, in turn promoting a cascade of splicing alterations to affect the maintenance of genomic, chromatin, and telomere integrity (see text for details).

While alterations in alternative splicing are likely contributing to aging, it is unclear what events initially trigger the change in activity of splicing regulators. Relevant to this question are recent studies in human cells that have uncovered links between telomere function and gene expression. Specifically, chromosome looping allows long telomeres to extend their effect on transcription as far as 10 Mb, a process termed Telomere Position‐Effect On Long Distance (TPE‐OLD) (Robin *et al*., 2014). As telomeres shorten during aging, TPE‐OLD regulation becomes compromised, directly altering gene expression (Robin *et al*., 2015). If the looped‐out chromosome portions host genes encoding splicing regulators or factors that control their activity, the loss of TPE‐OLD may have a global impact on alternative splicing. A link between telomere function and the activity of splicing factors is supported by the observation that telomere dysfunction promotes the production of the *LMNA* splice variant progerin, which elicits senescence (Cao *et al*., 2011). In addition, mouse hematopoietic progenitor cells from telomerase‐deficient mice have reduced levels of U2AF2, SF3B1, and SRSF2 (Colla *et al*., 2015). Moreover, telomerase‐deficient and SRSF2‐deficient mice share defects in the splicing of transcripts encoding components involved in DNA repair, chromatin structure, and telomere maintenance (Colla *et al*., 2015). These observations can be integrated into a model whereby TPE‐OLD would be important for the activity of splicing factors such as SRSF2 (Fig. 9). Notably, the human and mouse *Srsf2* and *U2af2* genes are located less than 10 Mb from the telomeres on their respective chromosomes. In young cells, optimal telomere function would insure optimal SRSF2 expression, allowing correct splicing of transcripts encoding telomere maintenance, DNA repair, and chromatin factors. In cells of aging animals however, telomere erosion would lead to a disruption of TPE‐OLD and decreased levels of SRSF2 that would elicit aberrant splicing of components required for genome integrity. It is also notable that age‐related splicing changes occur in the same categories of transcripts that are affected by DNA damaging agents (i.e., DNA repair, chromatin, and RNA splicing) (Southworth *et al*., 2009; Harries *et al*., 2011; Tollervey *et al*., 2011; Shkreta & Chabot, 2015). While short telomeres not engaged in TPE‐OLD may activate the DDR, this pathway may be further stimulated by the downstream pre‐mRNA splicing changes. The resulting amplification of genomic stress would lead to senescence (or cancer, if the senescent or apoptotic programs are subfunctional). The stochastic nature of telomere erosion (Martin‐Ruiz *et al*., 2004) may create variation in TPE‐OLD between cells, possibly contributing to the age‐dependent increase in transcriptional variability observed between individual cells (Martinez‐Jimenez *et al*., 2017), a process that may also apply to alternative splicing control.

Although senescence and aging affect a wide‐range of biological processes, interventions that prevent or encourage the production of specific splice variants may help counteract the emergence of age‐related phenotypes and disorders. Splice‐switching antisense oligonucleotides (SSOs) that block or stimulate the use of a splice site are now used with success in animal models of human diseases and are being tested in clinical trials (Brosseau *et al*., 2014; Havens & Hastings, 2016). In principle, they could be used to prevent the production of pro‐senescence variants in p53, ING1, and other transcripts. A recent encouraging study used an SSO to shift the splicing of *ApoER2* and explore its contribution to AD in a mouse model: correction of *ApoER2* splicing improved synaptic function, learning, and memory (Hinrich *et al*., 2016). Alternatively, given that a large collection of apoptotic regulators produce functionally antagonistic splice variants, and that the apoptotic pathway is repressed in senescent cells (Wang, 1995), SSOs may serve to encourage the production of pro‐death variants in senescent cells to favor their elimination and thus prevent their deleterious contribution to aging tissues. Other anti‐aging tools may emerge from the screening of small molecules capable of modulating splicing (Salton & Misteli, 2016). Compounds targeting generic splicing factors, such as SF3B1, may find a use as anticancer agents. As compounds that control the activity of SR proteins are being identified (Shkreta *et al*., 2017; Sigala *et al*., 2017), their pro‐apoptotic or antisenescent activities will need to be investigated. Overall, while additional links between splicing and different facets of aging will continue to be uncovered, compounds that can modulate specific splicing events or relevant splicing programs may offer innovative approaches to correct or postpone age‐related disorders.

## Funding

The work in the laboratory of BC is supported by the Canadian Institutes of Health Research (grant MOP‐136948). BC is the Pierre C. Fournier Chair in Functional Genomics.

## Conflict of interest

None declared.

## References

[acel12646-bib-0001] Abad M , Moreno A , Palacios A , Narita M , Blanco F , Moreno‐Bueno G , Narita M , Palmero I (2011) The tumor suppressor ING1 contributes to epigenetic control of cellular senescence. Aging Cell 10, 158–171. 21078114 10.1111/j.1474-9726.2010.00651.x

[acel12646-bib-0002] Abdelmohsen K , Gorospe M (2015) Noncoding RNA control of cellular senescence. Wiley Interdiscip. Rev. RNA 6, 615–629. 26331977 10.1002/wrna.1297 PMC4562317

[acel12646-bib-0003] Abdelmohsen K , Panda AC , Kang M‐J , Guo R , Kim J , Grammatikakis I , Yoon J‐H , Dudekula DB , Noh JH , Yang X , Martindale JL , Gorospe M (2014) 7SL RNA represses p53 translation by competing with HuR. Nucleic Acids Res. 42, 10099–10111. 25123665 10.1093/nar/gku686 PMC4150789

[acel12646-bib-0004] Acosta JC , Banito A , Wuestefeld T , Georgilis A , Janich P , Morton JP , Athineos D , Kang T‐W , Lasitschka F , Andrulis M , Pascual G , Morris KJ , Khan S , Jin H , Dharmalingam G , Snijders AP , Carroll T , Capper D , Pritchard C , Inman GJ , Longerich T , Sansom OJ , Benitah SA , Zender L , Gil J (2013) A complex secretory program orchestrated by the inflammasome controls paracrine senescence. Nat. Cell Biol. 15, 978–990. 23770676 10.1038/ncb2784 PMC3732483

[acel12646-bib-0005] Allsopp RC , Vaziri H , Patterson C , Goldstein S , Younglai EV , Futcher AB , Greider CW , Harley CB (1992) Telomere length predicts replicative capacity of human fibroblasts. Proc. Natl Acad. Sci. USA 89, 10114–10118. 1438199 10.1073/pnas.89.21.10114 PMC50288

[acel12646-bib-0006] Altintas O , Park S , Lee S‐JV (2016) The role of insulin/IGF‐1 signaling in the longevity of model invertebrates, *C. elegans* and *D. melanogaster* . BMB Rep. 49, 81–92. 26698870 10.5483/BMBRep.2016.49.2.261 PMC4915121

[acel12646-bib-0007] Baar MP , Brandt RMC , Putavet DA , Klein JDD , Derks KWJ , Bourgeois BRM , Stryeck S , Rijksen Y , van Willigenburg H , Feijtel DA , van der Pluijm I , Essers J , van Cappellen WA , van IJcken WF , Houtsmuller AB , Pothof J , de Bruin RWF , Madl T , Hoeijmakers JHJ , Campisi J , de Keizer PLJ (2017) Targeted apoptosis of senescent cells restores tissue homeostasis in response to chemotoxicity and aging. Cell 169, 132–147.e16. 28340339 10.1016/j.cell.2017.02.031 PMC5556182

[acel12646-bib-0008] Baker DJ , Perez‐Terzic C , Jin F , Pitel K , Niederländer NJ , Jeganathan K , Yamada S , Reyes S , Rowe L , Hiddinga HJ , Eberhardt NL , Terzic A , van Deursen JM (2008) Opposing roles for p16Ink4a and p19Arf in senescence and ageing caused by BubR1 insufficiency. Nat. Cell Biol. 10, 825–836. 18516091 10.1038/ncb1744 PMC2594014

[acel12646-bib-0009] Baker DJ , Wijshake T , Tchkonia T , LeBrasseur NK , Childs BG , van de Sluis B , Kirkland JL , van Deursen JM (2011) Clearance of p16Ink4a‐positive senescent cells delays ageing‐associated disorders. Nature 479, 232–236. 22048312 10.1038/nature10600 PMC3468323

[acel12646-bib-0010] Baker DJ , Childs BG , Durik M , Wijers ME , Sieben CJ , Zhong JA , Saltness R , Jeganathan KB , Verzosa GC , Pezeshki A , Khazaie K , Miller JD , van Deursen JM (2016) Naturally occurring p16Ink4a‐positive cells shorten healthy lifespan. Nature 530, 184–189. 26840489 10.1038/nature16932 PMC4845101

[acel12646-bib-0011] Beausejour CM (2003) Reversal of human cellular senescence: roles of the p53 and p16 pathways. EMBO J. 22, 4212–4222. 12912919 10.1093/emboj/cdg417 PMC175806

[acel12646-bib-0012] Bellón T , Corbí A , Lastres P , Calés C , Cebrián M , Vera S , Cheifetz S , Massague J , Letarte M , Bernabéu C (1993) Identification and expression of two forms of the human transforming growth factor‐beta‐binding protein endoglin with distinct cytoplasmic regions. Eur. J. Immunol. 23, 2340–2345. 8370410 10.1002/eji.1830230943

[acel12646-bib-0013] Benson EK , Lee SW , Aaronson SA (2010) Role of progerin‐induced telomere dysfunction in HGPS premature cellular senescence. J. Cell Sci. 123, 2605–2612. 20605919 10.1242/jcs.067306 PMC2908049

[acel12646-bib-0014] Bernabeu C , Conley BA , Vary CPH (2007) Novel biochemical pathways of endoglin in vascular cell physiology. J. Cell. Biochem. 102, 1375–1388. 17975795 10.1002/jcb.21594 PMC2199238

[acel12646-bib-0015] Binda O , Nassif C , Branton PE (2008) SIRT1 negatively regulates HDAC1‐dependent transcriptional repression by the RBP1 family of proteins. Oncogene 27, 3384–3392. 18193082 10.1038/sj.onc.1211014

[acel12646-bib-0016] Blanco FJ , Bernabeu C (2011) Alternative splicing factor or splicing factor‐2 plays a key role in intron retention of the endoglin gene during endothelial senescence. Aging Cell 10, 896–907. 21668763 10.1111/j.1474-9726.2011.00727.x

[acel12646-bib-0017] Blanco FJ , Grande MT , Langa C , Oujo B , Velasco S , Rodriguez‐Barbero A , Perez‐Gomez E , Quintanilla M , Lopez‐Novoa JM , Bernabeu C (2008) S‐endoglin expression is induced in senescent endothelial cells and contributes to vascular pathology. Circ. Res. 103, 1383–1392. 18974388 10.1161/CIRCRESAHA.108.176552

[acel12646-bib-0018] Bodnar AG , Ouellette M , Frolkis M , Holt SE , Chiu CP , Morin GB , Harley CB , Shay JW , Lichtsteiner S , Wright WE (1998) Extension of life‐span by introduction of telomerase into normal human cells. Science 279, 349–352. 9454332 10.1126/science.279.5349.349

[acel12646-bib-0019] Braidy N , Guillemin GJ , Mansour H , Chan‐Ling T , Poljak A , Grant R (2011) Age related changes in NAD+ metabolism oxidative stress and sirt1 activity in wistar rats. PLoS ONE 6, e19194. 10.1371/journal.pone.0019194 PMC3082551 21541336

[acel12646-bib-0020] Brandes R , Fleming I , Busse R (2005) Endothelial aging. Cardiovasc. Res. 66, 286–294. 15820197 10.1016/j.cardiores.2004.12.027

[acel12646-bib-0021] Brassard JA , Fekete N , Garnier A , Hoesli CA (2016) Hutchinson‐Gilford progeria syndrome as a model for vascular aging. Biogerontology 17, 129–145. 26330290 10.1007/s10522-015-9602-z

[acel12646-bib-0022] Brooks SV , Faulkner JA (1990) Contraction‐induced injury: recovery of skeletal muscles in young and old mice. Am. J. Physiol. 258, C436–C442. 2316632 10.1152/ajpcell.1990.258.3.C436

[acel12646-bib-0023] Brosseau J‐P , Lucier J‐F , Lamarche A‐A , Shkreta L , Gendron D , Lapointe E , Thibault P , Paquet É , Perreault J‐P , Abou Elela S , Chabot B (2014) Redirecting splicing with bifunctional oligonucleotides. Nucleic Acids Res. 42, e40. 24375754 10.1093/nar/gkt1287 PMC3973305

[acel12646-bib-0024] Brown VA , Patel KR , Viskaduraki M , Crowell JA , Perloff M , Booth TD , Vasilinin G , Sen A , Schinas AM , Piccirilli G , Brown K , Steward WP , Gescher AJ , Brenner DE (2010) Repeat dose study of the cancer chemopreventive agent resveratrol in healthy volunteers: safety, pharmacokinetics, and effect on the insulin‐like growth factor axis. Cancer Res. 70, 9003–9011. 20935227 10.1158/0008-5472.CAN-10-2364 PMC2982884

[acel12646-bib-0025] Burns A , Iliffe S (2009) Alzheimer's disease. BMJ 338, b158. 19196745 10.1136/bmj.b158

[acel12646-bib-0026] Burtner CR , Kennedy BK (2010) Progeria syndromes and ageing: what is the connection? Nat. Rev. Mol. Cell Biol. 11, 567–578. 20651707 10.1038/nrm2944

[acel12646-bib-0027] Busch A , Hertel KJ (2012) Evolution of SR protein and hnRNP splicing regulatory factors. Wiley Interdiscip. Rev. RNA 3, 1–12. 21898828 10.1002/wrna.100 PMC3235224

[acel12646-bib-0028] Cadet J , Douki T , Ravanat J‐L , Di Mascio P (2009) Sensitized formation of oxidatively generated damage to cellular DNA by UVA radiation. Photochem. Photobiol. Sci. 8, 903–911. 19582264 10.1039/b905343n

[acel12646-bib-0029] Campisi J (2004) Fragile fugue: p53 in aging, cancer and IGF signaling. Nat. Med. 10, 231–232. 14991040 10.1038/nm0304-231

[acel12646-bib-0030] Campisi J (2013) Aging, cellular senescence, and cancer. Annu. Rev. Physiol. 75, 685–705. 23140366 10.1146/annurev-physiol-030212-183653 PMC4166529

[acel12646-bib-0031] Candelario J , Sudhakar S , Navarro S , Reddy S , Comai L (2008) Perturbation of wild‐type lamin A metabolism results in a progeroid phenotype. Aging Cell 7, 355–367. 18363904 10.1111/j.1474-9726.2008.00393.x PMC2527236

[acel12646-bib-0032] Cao K , Blair CD , Faddah DA , Kieckhaefer JE , Olive M , Erdos MR , Nabel EG , Collins FS (2011) Progerin and telomere dysfunction collaborate to trigger cellular senescence in normal human fibroblasts. J. Clin. Invest. 121, 2833–2844. 21670498 10.1172/JCI43578 PMC3223819

[acel12646-bib-0033] Chabot B , Shkreta L (2016) Defective control of pre–messenger RNA splicing in human disease. J. Cell Biol. 212, 13–27. 26728853 10.1083/jcb.201510032 PMC4700483

[acel12646-bib-0034] Chami L , Checler F (2012) BACE1 is at the crossroad of a toxic vicious cycle involving cellular stress and β‐amyloid production in Alzheimer's disease. Mol. Neurodegener. 7, 52. 23039869 10.1186/1750-1326-7-52 PMC3507664

[acel12646-bib-0035] Cheng H‐L , Mostoslavsky R , Saito S , Manis JP , Gu Y , Patel P , Bronson R , Appella E , Alt FW , Chua KF (2003) Developmental defects and p53 hyperacetylation in Sir2 homolog (SIRT1)‐deficient mice. Proc. Natl Acad. Sci. USA 100, 10794–10799. 12960381 10.1073/pnas.1934713100 PMC196882

[acel12646-bib-0036] Cheung KJ , Bush JA , Jia W , Li G (2000) Expression of the novel tumour suppressor p33(ING1)is independent of p53. Br. J. Cancer 83, 1468–1472. 11076655 10.1054/bjoc.2000.1464 PMC2363422

[acel12646-bib-0037] Citron M , Teplow DB , Selkoe DJ (1995) Generation of amyloid beta protein from its precursor is sequence specific. Neuron 14, 661–670. 7695913 10.1016/0896-6273(95)90323-2

[acel12646-bib-0038] Coles AH , Liang H , Zhu Z , Marfella CGA , Kang J , Imbalzano AN , Jones SN (2007) Deletion of p37Ing1 in mice reveals a p53‐independent role for Ing1 in the suppression of cell proliferation, apoptosis, and tumorigenesis. Cancer Res. 67, 2054–2061. 17332334 10.1158/0008-5472.CAN-06-3558 PMC2872148

[acel12646-bib-0039] Colla S , Ong DST , Ogoti Y , Marchesini M , Mistry NA , Clise‐Dwyer K , Ang SA , Storti P , Viale A , Giuliani N , Ruisaard K , Ganan Gomez I , Bristow CA , Estecio M , Weksberg DC , Ho YW , Hu B , Genovese G , Pettazzoni P , Multani AS , Jiang S , Hua S , Ryan MC , Carugo A , Nezi L , Wei Y , Yang H , D'Anca M , Zhang L , Gaddis S , Gong T , Horner JW , Heffernan TP , Jones P , Cooper LJN , Liang H , Kantarjian H , Wang YA , Chin L , Bueso‐Ramos C , Garcia‐Manero G , DePinho RA (2015) Telomere dysfunction drives aberrant hematopoietic differentiation and myelodysplastic syndrome. Cancer Cell 27, 644–657. 25965571 10.1016/j.ccell.2015.04.007 PMC4596059

[acel12646-bib-0040] Colman RJ , Anderson RM , Johnson SC , Kastman EK , Kosmatka KJ , Beasley TM , Allison DB , Cruzen C , Simmons HA , Kemnitz JW , Richard W (2009) Caloric restriction delays disease onset and mortality in rhesus monkeys. Science 325, 201–204. 19590001 10.1126/science.1173635 PMC2812811

[acel12646-bib-0041] Coolican SA , Samuel DS , Ewton DZ , McWade FJ , Florini JR (1997) The mitogenic and myogenic actions of insulin‐like growth factors utilize distinct signaling pathways. J. Biol. Chem. 272, 6653–6662. 9045696 10.1074/jbc.272.10.6653

[acel12646-bib-0042] Coppé J‐P , Patil CK , Rodier F , Sun Y , Muñoz DP , Goldstein J , Nelson PS , Desprez P‐Y , Campisi J (2008) Senescence‐associated secretory phenotypes reveal cell‐nonautonomous functions of oncogenic RAS and the p53 tumor suppressor. PLoS Biol. 6, 2853–2868. 19053174 10.1371/journal.pbio.0060301 PMC2592359

[acel12646-bib-0043] Coppedè F , Migliore L (2010) DNA repair in premature aging disorders and neurodegeneration. Curr. Aging Sci. 3, 3–19. 20298165 10.2174/1874609811003010003

[acel12646-bib-0044] Courtois S , Verhaegh G , North S , Luciani M‐G , Lassus P , Hibner U , Oren M , Hainaut P (2002) DeltaN‐p53, a natural isoform of p53 lacking the first transactivation domain, counteracts growth suppression by wild‐type p53. Oncogene 21, 6722–6728. 12360399 10.1038/sj.onc.1205874

[acel12646-bib-0045] Courtois‐Cox S , Jones SL , Cichowski K (2008) Many roads lead to oncogene‐induced senescence. Oncogene 27, 2801–2809. 18193093 10.1038/sj.onc.1210950

[acel12646-bib-0046] Daguenet E , Dujardin G , Valcarcel J (2015) The pathogenicity of splicing defects: mechanistic insights into pre‐mRNA processing inform novel therapeutic approaches. EMBO Rep. 16, 1640–1655. 26566663 10.15252/embr.201541116 PMC4693517

[acel12646-bib-0047] De Sandre‐Giovannoli A , Bernard R , Cau P , Navarro C , Amiel J , Boccaccio I , Lyonnet S , Stewart CL , Munnich A , Le Merrer M , Lévy N (2003) Lamin a truncation in Hutchinson‐Gilford progeria. Science 300, 2055. 12702809 10.1126/science.1084125

[acel12646-bib-0048] Dechat T , Pfleghaar K , Sengupta K , Shimi T , Shumaker DK , Solimando L , Goldman RD (2008) Nuclear lamins: major factors in the structural organization and function of the nucleus and chromatin. Genes Dev. 22, 832–853. 18381888 10.1101/gad.1652708 PMC2732390

[acel12646-bib-0049] Dechat T , Adam SA , Goldman RD (2009) Nuclear lamins and chromatin: when structure meets function. Adv. Enzyme Regul. 49, 157–166. 19154754 10.1016/j.advenzreg.2008.12.003 PMC3253622

[acel12646-bib-0050] van Deursen JM (2014) The role of senescent cells in ageing. Nature 509, 439–446. 24848057 10.1038/nature13193 PMC4214092

[acel12646-bib-0051] Díaz‐Hernández JI , Sebastián‐Serrano Á , Gómez‐Villafuertes R , Díaz‐Hernández M , Miras‐Portugal MT (2015) Age‐related nuclear translocation of P2X6 subunit modifies splicing activity interacting with splicing factor 3A1. PLoS ONE 10, e0123121. 25874565 10.1371/journal.pone.0123121 PMC4395284

[acel12646-bib-0052] ten Dijke P , Arthur HM (2007) Extracellular control of TGFβ signalling in vascular development and disease. Nat. Rev. Mol. Cell Biol. 8, 857–869. 17895899 10.1038/nrm2262

[acel12646-bib-0053] Dimri GP , Lee X , Basile G , Acosta M , Scott G , Roskelley C , Medrano EE , Linskens M , Rubelj I , Pereira‐Smith O (1995) A biomarker that identifies senescent human cells in culture and in aging skin *in vivo* . Proc. Natl Acad. Sci. USA 92, 9363–9367. 7568133 10.1073/pnas.92.20.9363 PMC40985

[acel12646-bib-0054] Dlamini Z , Tshidino S , Hull R (2015) Abnormalities in alternative splicing of apoptotic genes and cardiovascular diseases. Int. J. Mol. Sci. 16, 27171–27190. 26580598 10.3390/ijms161126017 PMC4661875

[acel12646-bib-0055] Donehower LA , Harvey M , Slagle BL , McArthur MJ , Montgomery CA , Butel JS , Bradley A (1992) Mice deficient for p53 are developmentally normal but susceptible to spontaneous tumours. Nature 356, 215–221. 1552940 10.1038/356215a0

[acel12646-bib-0056] Düwel A , Eleno N , Jerkic M , Arevalo M , Bolaños JP , Bernabeu C , López‐Novoa JM (2006) Reduced tumor growth and angiogenesis in endoglin‐haploinsufficient mice. Tumor Biol. 28, 1–8. 10.1159/000097040 17108712

[acel12646-bib-0057] Eisenberg T , Knauer H , Schauer A , Büttner S , Ruckenstuhl C , Carmona‐Gutierrez D , Ring J , Schroeder S , Magnes C , Antonacci L , Fussi H , Deszcz L , Hartl R , Schraml E , Criollo A , Megalou E , Weiskopf D , Laun P , Heeren G , Breitenbach M , Grubeck‐Loebenstein B , Herker E , Fahrenkrog B , Fröhlich K‐U , Sinner F , Tavernarakis N , Minois N , Kroemer G , Madeo F (2009) Induction of autophagy by spermidine promotes longevity. Nat. Cell Biol. 11, 1305–1314. 19801973 10.1038/ncb1975

[acel12646-bib-0058] Eriksson M , Brown WT , Gordon LB , Glynn MW , Singer J , Scott L , Erdos MR , Robbins CM , Moses TY , Berglund P , Dutra A , Pak E , Durkin S , Csoka AB , Boehnke M , Glover TW , Collins FS (2003) Recurrent *de novo* point mutations in lamin A cause Hutchinson‐Gilford progeria syndrome. Nature 423, 293–298. 12714972 10.1038/nature01629 PMC10540076

[acel12646-bib-0059] Feige JN , Johan A (2008) Transcriptional targets of sirtuins in the coordination of mammalian physiology. Curr. Opin. Cell Biol. 20, 303–309. 18468877 10.1016/j.ceb.2008.03.012 PMC2447870

[acel12646-bib-0060] Feng X , Hara Y , Riabowol KT (2002) Different HATS of the ING1 gene family. Trends Cell Biol. 12, 532–538. 12446115 10.1016/s0962-8924(02)02391-7

[acel12646-bib-0061] Firestein R , Blander G , Michan S , Oberdoerffer P , Ogino S , Campbell J , Bhimavarapu A , Luikenhuis S , de Cabo R , Fuchs C , Hahn WC , Guarente LP , Sinclair DA (2008) The SIRT1 deacetylase suppresses intestinal tumorigenesis and colon cancer growth M. V. Blagosklonny, ed. PLoS ONE 3, e2020. 18414679 10.1371/journal.pone.0002020 PMC2289879

[acel12646-bib-0062] Fisette J‐F , Montagna DR , Mihailescu M‐R , Wolfe MS (2012) A G‐rich element forms a G‐quadruplex and regulates BACE1 mRNA alternative splicing. J. Neurochem. 121, 763–773. 22303960 10.1111/j.1471-4159.2012.07680.x PMC3342435

[acel12646-bib-0063] Flier JS , Underhill LH , Le Roith D (1997) Insulin‐like growth factors. N. Engl. J. Med. 336, 633–640. 9032050 10.1056/NEJM199702273360907

[acel12646-bib-0064] Foreman KE , Tang J (2003) Molecular mechanisms of replicative senescence in endothelial cells. Exp. Gerontol. 38, 1251–1257. 14698804 10.1016/j.exger.2003.09.005

[acel12646-bib-0065] Freund A , Laberge R‐M , Demaria M , Campisi J (2012) Lamin B1 loss is a senescence‐associated biomarker. Mol. Biol. Cell 23, 2066–2075. 22496421 10.1091/mbc.E11-10-0884 PMC3364172

[acel12646-bib-0066] Fu X‐D , Ares M (2014) Context‐dependent control of alternative splicing by RNA‐binding proteins. Nat. Rev. Genet. 15, 689–701. 25112293 10.1038/nrg3778 PMC4440546

[acel12646-bib-0067] Fujita K , Mondal AM , Horikawa I , Nguyen GH , Kumamoto K , Sohn JJ , Bowman ED , Mathe EA , Schetter AJ , Pine SR , Ji H , Vojtesek B , Bourdon J‐C , Lane DP , Harris CC (2009) p53 isoforms Δ133p53 and p53β are endogenous regulators of replicative cellular senescence. Nat. Cell Biol. 11, 1135–1142. 19701195 10.1038/ncb1928 PMC2802853

[acel12646-bib-0068] Fulco M , Schiltz RL , Iezzi S , King MT , Zhao P , Kashiwaya Y , Hoffman E , Veech RL , Sartorelli V (2003) Sir2 regulates skeletal muscle differentiation as a potential sensor of the redox state. Mol. Cell 12, 51–62. 12887892 10.1016/s1097-2765(03)00226-0

[acel12646-bib-0069] Gao W , Shen Z , Shang L , Wang X (2011) Upregulation of human autophagy‐initiation kinase ULK1 by tumor suppressor p53 contributes to DNA‐damage‐induced cell death. Cell Death Differ. 18, 1598–1607. 21475306 10.1038/cdd.2011.33 PMC3172118

[acel12646-bib-0070] Ghosh A , Stewart D , Matlashewski G (2004) Regulation of human p53 activity and cell localization by alternative splicing. Mol. Cell. Biol. 24, 7987–7997. 15340061 10.1128/MCB.24.18.7987-7997.2004 PMC515058

[acel12646-bib-0071] Giulietti M , Piva F , D'Antonio M , D'Onorio De Meo P , Paoletti D , Castrignano T , D'Erchia AM , Picardi E , Zambelli F , Principato G , Pavesi G , Pesole G (2013) SpliceAid‐F: a database of human splicing factors and their RNA‐binding sites. Nucleic Acids Res. 41, D125–D131. 23118479 10.1093/nar/gks997 PMC3531144

[acel12646-bib-0072] Goldman RD , Gruenbaum Y , Moir RD , Shumaker DK , Spann TP (2002) Nuclear lamins: building blocks of nuclear architecture. Genes Dev. 16, 533–547. 11877373 10.1101/gad.960502

[acel12646-bib-0073] Goldspink G (2005) Research on mechano growth factor: its potential for optimising physical training as well as misuse in doping. Br. J. Sports Med. 39, 787–788. 16244184 10.1136/bjsm.2004.015826 PMC1725070

[acel12646-bib-0074] Gruner H , Cortés‐López M , Cooper DA , Bauer M , Miura P (2016) CircRNA accumulation in the aging mouse brain. Sci. Rep. 6, 38907. 27958329 10.1038/srep38907 PMC5153657

[acel12646-bib-0075] Guarente L (2007) Sirtuins in aging and disease. Cold Spring Harb. Symp. Quant. Biol. 72, 483–488. 18419308 10.1101/sqb.2007.72.024

[acel12646-bib-0076] Guérillon C , Larrieu D , Pedeux R (2013) ING1 and ING2: multifaceted tumor suppressor genes. Cell. Mol. Life Sci. 70, 3753–3772. 23412501 10.1007/s00018-013-1270-z PMC11113716

[acel12646-bib-0077] Günes C , Rudolph KL (2012) Telomere dysfunction puts the brakes on oncogene‐induced cancers. EMBO J. 31, 2833–2834. 22643218 10.1038/emboj.2012.162 PMC3395100

[acel12646-bib-0078] Guo W , Schafer S , Greaser ML , Radke MH , Liss M , Govindarajan T , Maatz H , Schulz H , Li S , Parrish AM , Dauksaite V , Vakeel P , Klaassen S , Gerull B , Thierfelder L , Regitz‐Zagrosek V , Hacker TA , Saupe KW , Dec GW , Ellinor PT , MacRae CA , Spallek B , Fischer R , Perrot A , Özcelik C , Saar K , Hubner N , Gotthardt M (2012) RBM20, a gene for hereditary cardiomyopathy, regulates titin splicing. Nat. Med. 18, 766–773. 22466703 10.1038/nm.2693 PMC3569865

[acel12646-bib-0079] Hameed M , Orrell RW , Cobbold M , Goldspink G , Harridge SDR (2003) Expression of IGF‐I splice variants in young and old human skeletal muscle after high resistance exercise. J. Physiol. 547, 247–254. 12562960 10.1113/jphysiol.2002.032136 PMC2342624

[acel12646-bib-0080] Harries LW , Hernandez D , Henley W , Wood AR , Holly AC , Bradley‐Smith RM , Yaghootkar H , Dutta A , Murray A , Frayling TM , Guralnik JM , Bandinelli S , Singleton A , Ferrucci L , Melzer D (2011) Human aging is characterized by focused changes in gene expression and deregulation of alternative splicing. Aging Cell 10, 868–878. 21668623 10.1111/j.1474-9726.2011.00726.x PMC3173580

[acel12646-bib-0081] Havens MA , Hastings ML (2016) Splice‐switching antisense oligonucleotides as therapeutic drugs. Nucleic Acids Res. 44, 6549–6563. 27288447 10.1093/nar/gkw533 PMC5001604

[acel12646-bib-0082] Hayflick L , Moorhead PS (1961) The serial cultivation of human diploid cell strains. Exp. Cell Res. 25, 585–621. 13905658 10.1016/0014-4827(61)90192-6

[acel12646-bib-0083] van Heemst D , Mooijaart SP , Beekman M , Schreuder J , de Craen AJM , Brandt BW , Eline Slagboom P , Westendorp RGJ (2005) Variation in the human TP53 gene affects old age survival and cancer mortality. Exp. Gerontol. 40, 11–15. 15732191 10.1016/j.exger.2004.10.001

[acel12646-bib-0084] Heintz C , Doktor TK , Lanjuin A , Escoubas CC , Zhang Y , Weir HJ , Dutta S , Silva‐García CG , Bruun GH , Morantte I , Hoxhaj G , Manning BD , Andresen BS , Mair WB (2016) Splicing factor 1 modulates dietary restriction and TORC1 pathway longevity in *C. elegans* . Nature 541, 102–106. 27919065 10.1038/nature20789 PMC5361225

[acel12646-bib-0085] Hennekam RCM (2006) Hutchinson‐Gilford progeria syndrome: review of the phenotype. Am. J. Med. Genet. Part A 140A, 2603–2624. 10.1002/ajmg.a.31346 16838330

[acel12646-bib-0086] Herbig U , Ferreira M , Condel L , Carey D , Sedivy JM (2006) Cellular senescence in aging primates. Science 311, 1257. 16456035 10.1126/science.1122446

[acel12646-bib-0087] Higashi Y , Quevedo HC , Tiwari S , Sukhanov S , Shai S‐Y , Anwar A , Delafontaine P (2014) Interaction between insulin‐like growth factor‐1 and atherosclerosis and vascular aging. Front. Horm. Res. 43, 107–124. 24943302 10.1159/000360571 PMC4199335

[acel12646-bib-0088] Hinrich AJ , Jodelka FM , Chang JL , Brutman D , Bruno AM , Briggs CA , James BD , Stutzmann GE , Bennett DA , Miller SA , Rigo F , Marr RA , Hastings ML (2016) Therapeutic correction of ApoER2 splicing in Alzheimer's disease mice using antisense oligonucleotides. EMBO Mol. Med. 8, 328–345. 26902204 10.15252/emmm.201505846 PMC4818756

[acel12646-bib-0089] Hisama FM , Lessel D , Leistritz D , Friedrich K , McBride KL , Pastore MT , Gottesman GS , Saha B , Martin GM , Kubisch C , Oshima J (2011) Coronary artery disease in a Werner syndrome‐like form of progeria characterized by low levels of progerin, a splice variant of lamin A. Am. J. Med. Genet. A 155A, 3002–3006. 22065502 10.1002/ajmg.a.34336 PMC4679285

[acel12646-bib-0090] Holly AC , Melzer D , Pilling LC , Fellows AC , Tanaka T , Ferrucci L , Harries LW (2013) Changes in splicing factor expression are associated with advancing age in man. Mech. Ageing Dev. 134, 356–366. 23747814 10.1016/j.mad.2013.05.006 PMC5863542

[acel12646-bib-0091] Howard JM , Sanford JR (2015) The RNAissance family: SR proteins as multifaceted regulators of gene expression. Wiley Interdiscip. Rev. RNA 6, 93–110. 25155147 10.1002/wrna.1260 PMC4268343

[acel12646-bib-0092] Hsiao K , Chapman P , Nilsen S , Eckman C , Harigaya Y , Younkin S , Yang F , Cole G (1996) Correlative memory deficits, Abeta elevation, and amyloid plaques in transgenic mice. Science 274, 99–102. 8810256 10.1126/science.274.5284.99

[acel12646-bib-0093] Huang S , Chen L , Libina N , Janes J , Martin GM , Campisi J , Oshima J (2005) Correction of cellular phenotypes of Hutchinson‐Gilford Progeria cells by RNA interference. Hum. Genet. 118, 444–450. 16208517 10.1007/s00439-005-0051-7

[acel12646-bib-0094] Huot M‐É , Vogel G , Zabarauskas A , Ngo CT‐A , Coulombe‐Huntington J , Majewski J , Richard S (2012) The Sam68 STAR RNA‐binding protein regulates mTOR alternative splicing during adipogenesis. Mol. Cell 46, 187–199. 22424772 10.1016/j.molcel.2012.02.007

[acel12646-bib-0095] Hutchison CJ (2002) Lamins: building blocks or regulators of gene expression? Nat. Rev. Mol. Cell Biol. 3, 848–858. 12415302 10.1038/nrm950

[acel12646-bib-0096] Ikehata H , Ono T (2011) The mechanisms of UV mutagenesis. J. Radiat. Res. 52, 115–125. 21436607 10.1269/jrr.10175

[acel12646-bib-0097] Jeyapalan JC , Ferreira M , Sedivy JM , Herbig U (2007) Accumulation of senescent cells in mitotic tissue of aging primates. Mech. Ageing Dev. 128, 36–44. 17116315 10.1016/j.mad.2006.11.008 PMC3654105

[acel12646-bib-0098] Jin Y , Yang Y , Zhang P (2011) New insights into RNA secondary structure in the alternative splicing of pre‐mRNAs. RNA Biol. 8, 450–457. 21558794 10.4161/rna.8.3.15388

[acel12646-bib-0099] Kabra N , Li Z , Chen L , Li B , Zhang X , Wang C , Yeatman T , Coppola D , Chen J (2009) SirT1 is an inhibitor of proliferation and tumor formation in colon cancer. J. Biol. Chem. 284, 18210–18217. 19433578 10.1074/jbc.M109.000034 PMC2709385

[acel12646-bib-0100] Kaeberlein M , McVey M , Guarente L (1999) The SIR2/3/4 complex and SIR2 alone promote longevity in *Saccharomyces cerevisiae* by two different mechanisms. Genes Dev. 13, 2570–2580. 10521401 10.1101/gad.13.19.2570 PMC317077

[acel12646-bib-0101] Kamijo T , Weber JD , Zambetti G , Zindy F , Roussel MF , Sherr CJ (1998) Functional and physical interactions of the ARF tumor suppressor with p53 and Mdm2. Proc. Natl Acad. Sci. USA 95, 8292–8297. 9653180 10.1073/pnas.95.14.8292 PMC20969

[acel12646-bib-0102] Kandalepas PC , Vassar R (2014) The normal and pathologic roles of the Alzheimer's β‐secretase, BACE1. Curr. Alzheimer Res. 11, 441–449. 24893886 10.2174/1567205011666140604122059 PMC4401991

[acel12646-bib-0103] Karlseder J (2002) Senescence induced by altered telomere state, not telomere loss. Science 295, 2446–2449. 11923537 10.1126/science.1069523

[acel12646-bib-0104] Kataoka H , Bonnefin P , Vieyra D , Feng X , Hara Y , Miura Y , Joh T , Nakabayashi H , Vaziri H , Harris CC , Riabowol K (2003) ING1 represses transcription by direct DNA binding and through effects on p53. Cancer Res. 63, 5785–5792. 14522900

[acel12646-bib-0105] Kelemen O , Convertini P , Zhang Z , Wen Y , Shen M , Falaleeva M , Stamm S (2013) Function of alternative splicing. Gene 514, 1–30. 22909801 10.1016/j.gene.2012.07.083 PMC5632952

[acel12646-bib-0106] Kichina JV , Zeremski M , Aris L , Gurova KV , Walker E , Franks R , Nikitin AY , Kiyokawa H , Gudkov AV (2006) Targeted disruption of the mouse ing1 locus results in reduced body size, hypersensitivity to radiation and elevated incidence of lymphomas. Oncogene 25, 857–866. 16170338 10.1038/sj.onc.1209118

[acel12646-bib-0107] Kim H‐J , Oh G‐S , Choe S‐K , Kwak TH , Park R , So H‐S (2014) NAD(+) metabolism in age‐related hearing loss. Aging Dis. 5, 150–159. 24729940 10.14336/AD.2014.0500150 PMC3966673

[acel12646-bib-0108] Kirkwood TBL (2005) Understanding the odd science of aging. Cell 120, 437–447. 15734677 10.1016/j.cell.2005.01.027

[acel12646-bib-0109] Kirkwood TBL , Holliday R (1979) The evolution of ageing and longevity. Proc. R. Soc. B Biol. Sci. 205, 531–546. 42059 10.1098/rspb.1979.0083

[acel12646-bib-0110] Koffie RM , Hyman BT , Spires‐Jones TL (2011) Alzheimer's disease: synapses gone cold. Mol. Neurodegener. 6, 63. 21871088 10.1186/1750-1326-6-63 PMC3178498

[acel12646-bib-0111] Kreiling JA , Tamamori‐Adachi M , Sexton AN , Jeyapalan JC , Munoz‐Najar U , Peterson AL , Manivannan J , Rogers ES , Pchelintsev NA , Adams PD , Sedivy JM (2011) Age‐associated increase in heterochromatic marks in murine and primate tissues. Aging Cell 10, 292–304. 21176091 10.1111/j.1474-9726.2010.00666.x PMC3079313

[acel12646-bib-0112] Krizhanovsky V , Yon M , Dickins RA , Hearn S , Simon J , Miething C , Yee H , Zender L , Lowe SW (2008) Senescence of activated stellate cells limits liver fibrosis. Cell 134, 657–667. 18724938 10.1016/j.cell.2008.06.049 PMC3073300

[acel12646-bib-0113] Kuilman T , Michaloglou C , Mooi WJ , Peeper DS (2010) The essence of senescence. Genes Dev. 24, 2463–2479. 21078816 10.1101/gad.1971610 PMC2975923

[acel12646-bib-0114] Lebrin F , Goumans M‐J , Jonker L , Carvalho RLC , Valdimarsdottir G , Thorikay M , Mummery C , Arthur HM , ten Dijke P (2004) Endoglin promotes endothelial cell proliferation and TGF‐beta/ALK1 signal transduction. EMBO J. 23, 4018–4028. 15385967 10.1038/sj.emboj.7600386 PMC524335

[acel12646-bib-0115] Ledford H (2011) Longevity genes challenged. Nature https://doi.org/10.1038/news.2011.549.

[acel12646-bib-0116] Lee Y , Rio DC (2015) Mechanisms and regulation of alternative pre‐mRNA splicing. Annu. Rev. Biochem. 84, 291–323. 25784052 10.1146/annurev-biochem-060614-034316 PMC4526142

[acel12646-bib-0117] Lee BP , Pilling LC , Emond F , Flurkey K , Harrison DE , Yuan R , Peters LL , Kuchel GA , Ferrucci L , Melzer D , Harries LW (2016) Changes in the expression of splicing factor transcripts and variations in alternative splicing are associated with lifespan in mice and humans. Aging Cell 15, 903–913. 27363602 10.1111/acel.12499 PMC5013025

[acel12646-bib-0118] LeWinter MM , Granzier HL (2013) Titin is a major human disease gene. Circulation 127, 938–944. 23439446 10.1161/CIRCULATIONAHA.112.139717 PMC3594684

[acel12646-bib-0119] Li N , Li Q , Cao X , Zhao G , Xue L , Tong T (2011) The tumor suppressor p33ING1b upregulates p16INK4a expression and induces cellular senescence. FEBS Lett. 585, 3106–3112. 21896275 10.1016/j.febslet.2011.08.044

[acel12646-bib-0120] Li S , Guo W , Dewey CN , Greaser ML (2013) Rbm20 regulates titin alternative splicing as a splicing repressor. Nucleic Acids Res. 41, 2659–2672. 23307558 10.1093/nar/gks1362 PMC3575840

[acel12646-bib-0121] Liang C (2010) Negative regulation of autophagy. Cell Death Differ. 17, 1807–1815. 20865012 10.1038/cdd.2010.115 PMC3131090

[acel12646-bib-0122] Lin AW , Barradas M , Stone JC , van Aelst L , Serrano M , Lowe SW (1998) Premature senescence involving p53 and p16 is activated in response to constitutive MEK/MAPK mitogenic signaling. Genes Dev. 12, 3008–3019. 9765203 10.1101/gad.12.19.3008 PMC317198

[acel12646-bib-0123] Liu B , Ghosh S , Yang X , Zheng H , Liu X , Wang Z , Jin G , Zheng B , Kennedy BK , Suh Y , Kaeberlein M , Tryggvason K , Zhou Z (2012) Resveratrol rescues SIRT1‐dependent adult stem cell Decline and alleviates progeroid features in laminopathy‐based progeria. Cell Metab. 16, 738–750. 23217256 10.1016/j.cmet.2012.11.007

[acel12646-bib-0124] Lombard DB , Pletcher SD , Cantó C , Auwerx J (2011) Ageing: longevity hits a roadblock. Nature 477, 410–411. 21938058 10.1038/477410a

[acel12646-bib-0125] Lopez‐Mejia IC , Vautrot V , De Toledo M , Behm‐Ansmant I , Bourgeois CF , Navarro CL , Osorio FG , Freije JMP , Stevenin J , De Sandre‐Giovannoli A , Lopez‐Otin C , Levy N , Branlant C , Tazi J (2011) A conserved splicing mechanism of the LMNA gene controls premature aging. Hum. Mol. Genet. 20, 4540–4555. 21875900 10.1093/hmg/ddr385

[acel12646-bib-0126] López‐Otín C , Blasco MA , Partridge L , Serrano M , Kroemer G (2013) The hallmarks of aging. Cell 153, 1194–1217. 23746838 10.1016/j.cell.2013.05.039 PMC3836174

[acel12646-bib-0127] Lowe SW , Cepero E , Evan G (2004) Intrinsic tumour suppression. Nature 432, 307–315. 15549092 10.1038/nature03098

[acel12646-bib-0128] Luo J , Nikolaev AY , Imai S , Chen D , Su F , Shiloh A , Guarente L , Gu W (2001) Negative control of p53 by Sir2α promotes cell survival under stress. Cell 107, 137–148. 11672522 10.1016/s0092-8674(01)00524-4

[acel12646-bib-0129] Lynch CJ , Shah ZH , Allison SJ , Ahmed SU , Ford J , Warnock LJ , Li H , Serrano M , Milner J (2010) SIRT1 undergoes alternative splicing in a novel auto‐regulatory loop with p53 M. V. Blagosklonny, ed. PLoS ONE 5, e13502. 20975832 10.1371/journal.pone.0013502 PMC2958826

[acel12646-bib-0130] Maatz H , Jens M , Liss M , Schafer S , Heinig M , Kirchner M , Adami E , Rintisch C , Dauksaite V , Radke MH , Selbach M , Barton PJR , Cook SA , Rajewsky N , Gotthardt M , Landthaler M , Hubner N (2014) RNA‐binding protein RBM20 represses splicing to orchestrate cardiac pre‐mRNA processing. J. Clin. Invest. 124, 3419–3430. 24960161 10.1172/JCI74523 PMC4109538

[acel12646-bib-0131] Maier B , Gluba W , Bernier B , Turner T , Mohammad K , Guise T , Sutherland A , Thorner M , Scrable H (2004) Modulation of mammalian life span by the short isoform of p53. Genes Dev. 18, 306–319. 14871929 10.1101/gad.1162404 PMC338283

[acel12646-bib-0132] Martin E , Golunski E , Laing ST , Estrera AL , Sharina IG (2014) Alternative splicing impairs soluble guanylyl cyclase function in aortic aneurysm. Am. J. Physiol. Heart Circ. Physiol. 307, H1565–H1575. 25239802 10.1152/ajpheart.00222.2014 PMC4255009

[acel12646-bib-0133] Martinez‐Contreras R , Cloutier P , Shkreta L , Fisette J‐F , Revil T , Chabot B (2007) hnRNP proteins and splicing control. Adv. Exp. Med. Biol. 623, 123–147. 18380344 10.1007/978-0-387-77374-2_8

[acel12646-bib-0134] Martinez‐Jimenez CP , Eling N , Chen H‐C , Vallejos CA , Kolodziejczyk AA , Connor F , Stojic L , Rayner TF , Stubbington MJT , Teichmann SA , de la Roche M , Marioni JC , Odom DT (2017) Aging increases cell‐to‐cell transcriptional variability upon immune stimulation. Science 355, 1433–1436. 28360329 10.1126/science.aah4115 PMC5405862

[acel12646-bib-0135] Martin‐Ruiz C , Saretzki G , Petrie J , Ladhoff J , Jeyapalan J , Wei W , Sedivy J , von Zglinicki T (2004) Stochastic variation in telomere shortening rate causes heterogeneity of human fibroblast replicative life span. J. Biol. Chem. 279, 17826–17833. 14963037 10.1074/jbc.M311980200

[acel12646-bib-0136] Massudi H , Grant R , Braidy N , Guest J , Farnsworth B , Guillemin GJ (2012) Age‐associated changes in oxidative stress and NAD+ metabolism in human tissue M. Polymenis, ed. PLoS ONE 7, e42357. 22848760 10.1371/journal.pone.0042357 PMC3407129

[acel12646-bib-0137] Matera AG , Wang Z (2014) A day in the life of the spliceosome. Nat. Rev. Mol. Cell Biol. 15, 108–121. 24452469 10.1038/nrm3742 PMC4060434

[acel12646-bib-0138] Mazan‐Mamczarz K , Galbán S , López de Silanes I , Martindale JL , Atasoy U , Keene JD , Gorospe M (2003) RNA‐binding protein HuR enhances p53 translation in response to ultraviolet light irradiation. Proc. Natl Acad. Sci. USA 100, 8354–8359. 12821781 10.1073/pnas.1432104100 PMC166233

[acel12646-bib-0139] Mazin P , Xiong J , Liu X , Yan Z , Zhang X , Li M , He L , Somel M , Yuan Y , Phoebe Chen Y‐P , Li N , Hu Y , Fu N , Ning Z , Zeng R , Yang H , Chen W , Gelfand M , Khaitovich P (2013) Widespread splicing changes in human brain development and aging. Mol. Syst. Biol. 9, 633. 23340839 10.1038/msb.2012.67 PMC3564255

[acel12646-bib-0140] McBurney MW , Yang X , Jardine K , Hixon M , Boekelheide K , Webb JR , Lansdorp PM , Lemieux M (2003) The mammalian SIR2alpha protein has a role in embryogenesis and gametogenesis. Mol. Cell. Biol. 23, 38–54. 12482959 10.1128/MCB.23.1.38-54.2003 PMC140671

[acel12646-bib-0141] McClintock D , Ratner D , Lokuge M , Owens DM , Gordon LB , Collins FS , Djabali K (2007) The mutant form of Lamin A that causes Hutchinson‐Gilford progeria is a biomarker of cellular aging in human skin. PLoS ONE 2, e1269. 10.1371/journal.pone.0001269 PMC2092390 18060063

[acel12646-bib-0142] Medina MW , Gao F , Naidoo D , Rudel LL , Temel RE , McDaniel AL , Marshall SM , Krauss RM (2011) Coordinately regulated alternative splicing of genes involved in cholesterol biosynthesis and uptake Y. Xu, ed. PLoS ONE 6, e19420. 21559365 10.1371/journal.pone.0019420 PMC3084847

[acel12646-bib-0143] Merideth MA , Gordon LB , Clauss S , Sachdev V , Smith ACM , Perry MB , Brewer CC , Zalewski C , Kim HJ , Solomon B , Brooks BP , Gerber LH , Turner ML , Domingo DL , Hart TC , Graf J , Reynolds JC , Gropman A , Yanovski JA , Gerhard‐Herman M , Collins FS , Nabel EG , Cannon RO , Gahl WA , Introne WJ (2008) Phenotype and course of Hutchinson‐Gilford progeria syndrome. N. Engl. J. Med. 358, 592–604. 18256394 10.1056/NEJMoa0706898 PMC2940940

[acel12646-bib-0144] Minamino T , Komuro I (2007) Vascular cell senescence: contribution to atherosclerosis. Circ. Res. 100, 15–26. 17204661 10.1161/01.RES.0000256837.40544.4a

[acel12646-bib-0145] Mitchell SJ , Martin‐Montalvo A , Mercken EM , Palacios HH , Ward TM , Abulwerdi G , Minor RK , Vlasuk GP , Ellis JL , Sinclair DA , Dawson J , Allison DB , Zhang Y , Becker KG , Bernier M , de Cabo R (2014) The SIRT1 activator SRT1720 extends lifespan and improves health of mice fed a standard diet. Cell Rep. 6, 836–843. 24582957 10.1016/j.celrep.2014.01.031 PMC4010117

[acel12646-bib-0146] Morselli E , Maiuri MC , Markaki M , Megalou E , Pasparaki A , Palikaras K , Criollo A , Galluzzi L , Malik SA , Vitale I , Michaud M , Madeo F , Tavernarakis N , Kroemer G (2010) The life span‐prolonging effect of Sirtuin‐1 is mediated by autophagy. Autophagy 6, 186–188. 20023410 10.4161/auto.6.1.10817

[acel12646-bib-0147] Mowrer KR , Wolfe MS (2008) Promotion of BACE1 mRNA alternative splicing reduces amyloid beta‐peptide production. J. Biol. Chem. 283, 18694–18701. 18468996 10.1074/jbc.M801322200

[acel12646-bib-0148] Mowrer KR , Wolfe MS (2009) Identification of a cis‐acting element involved in the regulation of BACE1 mRNA alternative splicing. J. Neurochem. 109, 1008–1016. 19302194 10.1111/j.1471-4159.2009.06026.x

[acel12646-bib-0149] Mozaffarian D , Wu JHY (2011) Omega‐3 fatty acids and cardiovascular disease. J. Am. Coll. Cardiol. 58, 2047–2067. 22051327 10.1016/j.jacc.2011.06.063

[acel12646-bib-0150] Naftelberg S , Schor IE , Ast G , Kornblihtt AR (2015) Regulation of alternative splicing through coupling with transcription and chromatin structure. Annu. Rev. Biochem. 84, 165–198. 26034889 10.1146/annurev-biochem-060614-034242

[acel12646-bib-0151] Nardella C , Clohessy JG , Alimonti A , Pandolfi PP (2011) Pro‐senescence therapy for cancer treatment. Nat. Rev. Cancer 11, 503–511. 21701512 10.1038/nrc3057

[acel12646-bib-0152] Narita M , Nũnez S , Heard E , Narita M , Lin AW , Hearn SA , Spector DL , Hannon GJ , Lowe SW (2003) Rb‐mediated heterochromatin formation and silencing of E2F target genes during cellular senescence. Cell 113, 703–716. 12809602 10.1016/s0092-8674(03)00401-x

[acel12646-bib-0153] Nelson PT , Head E , Schmitt FA , Davis PR , Neltner JH , Jicha GA , Abner EL , Smith CD , Van Eldik LJ , Kryscio RJ , Scheff SW (2011) Alzheimer's disease is not “brain aging”: neuropathological, genetic, and epidemiological human studies. Acta Neuropathol. 121, 571–587. 21516511 10.1007/s00401-011-0826-y PMC3179861

[acel12646-bib-0154] Nelson G , Wordsworth J , Wang C , Jurk D , Lawless C , Martin‐Ruiz C , von Zglinicki T (2012) A senescent cell bystander effect: senescence‐induced senescence. Aging Cell 11, 345–349. 22321662 10.1111/j.1474-9726.2012.00795.x PMC3488292

[acel12646-bib-0155] Nemoto S , Fergusson MM , Finkel T (2005) SIRT1 functionally interacts with the metabolic regulator and transcriptional coactivator PGC‐1. J. Biol. Chem. 280, 16456–16460. 15716268 10.1074/jbc.M501485200

[acel12646-bib-0156] Nieto Moreno N , Giono LE , Cambindo Botto AE , Muñoz MJ , Kornblihtt AR (2015) Chromatin, DNA structure and alternative splicing. FEBS Lett. 589, 3370–3378. 26296319 10.1016/j.febslet.2015.08.002

[acel12646-bib-0157] Nikolich‐Zugich J (2008) Ageing and life‐long maintenance of T‐cell subsets in the face of latent persistent infections. Nat. Rev. Immunol. 8, 512–522. 18469829 10.1038/nri2318 PMC5573867

[acel12646-bib-0158] Onel K , Cordon‐Cardo C (2004) MDM2 and prognosis. Mol. Cancer Res. 2, 1–8. 14757840

[acel12646-bib-0159] Ørsted DD , Bojesen SE , Tybjaerg‐Hansen A , Nordestgaard BG (2007) Tumor suppressor p53 Arg72Pro polymorphism and longevity, cancer survival, and risk of cancer in the general population. J. Exp. Med. 204, 1295–1301. 17535973 10.1084/jem.20062476 PMC2118619

[acel12646-bib-0160] Owino V , Yang SY , Goldspink G (2001) Age‐related loss of skeletal muscle function and the inability to express the autocrine form of insulin‐like growth factor‐1 (MGF) in response to mechanical overload. FEBS Lett. 505, 259–263. 11566187 10.1016/s0014-5793(01)02825-3

[acel12646-bib-0161] Pan Q , Shai O , Lee LJ , Frey BJ , Blencowe BJ (2008) Deep surveying of alternative splicing complexity in the human transcriptome by high‐throughput sequencing. Nat. Genet. 40, 1413–1415. 18978789 10.1038/ng.259

[acel12646-bib-0162] Panasyuk G , Nemazanyy I , Zhyvoloup A , Filonenko V , Davies D , Robson M , Pedley RB , Waterfield M , Gout I (2009) mTOR splicing isoform promotes cell proliferation and tumorigenesis. J. Biol. Chem. 284, 30807–30814. 19726679 10.1074/jbc.M109.056085 PMC2781479

[acel12646-bib-0163] Panda AC , Abdelmohsen K , Gorospe M (2017a) SASP regulation by noncoding RNA. Mech. Ageing Dev. pii: S0047‐6374(16)30298‐6. https://doi.org/10.1016/j.mad.2017.05.004. [Epub ahead of print]. 10.1016/j.mad.2017.05.004 PMC5681880 28502821

[acel12646-bib-0164] Panda AC , Grammatikakis I , Kim KM , De S , Martindale JL , Munk R , Yang X , Abdelmohsen K , Gorospe M (2017b) Identification of senescence‐associated circular RNAs (SAC‐RNAs) reveals senescence suppressor CircPVT1. Nucleic Acids Res. 45, 4021–4035. 27928058 10.1093/nar/gkw1201 PMC5397146

[acel12646-bib-0165] Parrinello S , Coppe J‐P , Krtolica A , Campisi J (2005) Stromal‐epithelial interactions in aging and cancer: senescent fibroblasts alter epithelial cell differentiation. J. Cell Sci. 118, 485–496. 15657080 10.1242/jcs.01635 PMC4939801

[acel12646-bib-0166] Pehar M , O'Riordan KJ , Burns‐Cusato M , Andrzejewski ME , del Alcazar CG , Burger C , Scrable H , Puglielli L (2010) Altered longevity‐assurance activity of p53:p44 in the mouse causes memory loss, neurodegeneration and premature death. Aging Cell 9, 174–190. 20409077 10.1111/j.1474-9726.2010.00547.x PMC2848983

[acel12646-bib-0167] Pehar M , Ko MH , Li M , Scrable H , Puglielli L (2014) P44, the “longevity‐assurance” isoform of P53, regulates tau phosphorylation and is activated in an age‐dependent fashion. Aging Cell 13, 449–456. 24341977 10.1111/acel.12192 PMC4032616

[acel12646-bib-0168] Peña PV , Hom RA , Hung T , Lin H , Kuo AJ , Wong RPC , Subach OM , Champagne KS , Zhao R , Verkhusha VV , Li G , Gozani O , Kutateladze TG (2008) Histone H3K4me3 binding is required for the DNA repair and apoptotic activities of ING1 tumor suppressor. J. Mol. Biol. 380, 303–312. 18533182 10.1016/j.jmb.2008.04.061 PMC2576750

[acel12646-bib-0169] Pérez‐Gómez E , Eleno N , López‐Novoa JM , Ramirez JR , Velasco B , Letarte M , Bernabéu C , Quintanilla M (2005) Characterization of murine S‐endoglin isoform and its effects on tumor development. Oncogene 24, 4450–4461. 15806144 10.1038/sj.onc.1208644

[acel12646-bib-0170] Philippou A , Papageorgiou E , Bogdanis G , Halapas A , Sourla A , Maridaki M , Pissimissis N , Koutsilieris M (2009) Expression of IGF‐1 isoforms after exercise‐induced muscle damage in humans: characterization of the MGF E peptide actions *in vitro* . In Vivo 23, 567–576. 19567392

[acel12646-bib-0171] Raj N , Attardi LD (2013) Tumor suppression: p53 alters immune surveillance to restrain liver cancer. Curr. Biol. 23, R527–R530. 23787049 10.1016/j.cub.2013.04.076 PMC4077325

[acel12646-bib-0172] Ray PS , Grover R , Das S (2006) Two internal ribosome entry sites mediate the translation of p53 isoforms. EMBO Rep. 7, 404–410. 16440000 10.1038/sj.embor.7400623 PMC1456917

[acel12646-bib-0173] Reardon HT , Park WJ , Zhang J , Lawrence P , Kothapalli KSD , Brenna JT (2011) The polypyrimidine tract binding protein regulates desaturase alternative splicing and PUFA composition. J. Lipid Res. 52, 2279–2286. 21980057 10.1194/jlr.M019653 PMC3220295

[acel12646-bib-0174] Ressler S , Bartkova J , Niederegger H , Bartek J , Scharffetter‐Kochanek K , Jansen‐Durr P , Wlaschek M (2006) p16 INK4A is a robust *in vivo* biomarker of cellular aging in human skin. Aging Cell 5, 379–389. 16911562 10.1111/j.1474-9726.2006.00231.x

[acel12646-bib-0175] Ribera‐Casado JM (1999) Ageing and the cardiovascular system. Z. Gerontol. Geriatr. 32, 412–419. 10654379 10.1007/s003910050138

[acel12646-bib-0176] Richard S , Torabi N , Franco GV , Tremblay GA , Chen T , Vogel G , Morel M , Cléroux P , Forget‐Richard A , Komarova S , Tremblay ML , Li W , Li A , Gao YJ , Henderson JE (2005) Ablation of the Sam68 RNA binding protein protects mice from age‐related bone loss. PLoS Genet. 1, e74. 16362077 10.1371/journal.pgen.0010074 PMC1315279

[acel12646-bib-0177] Rizzacasa B , Morini E , Pucci S , Murdocca M , Novelli G , Amati F (2017) LOX‐1 and its splice variants: a new challenge for atherosclerosis and cancer‐targeted therapies. Int. J. Mol. Sci. 18, E290. 10.3390/ijms18020290 PMC5343826 28146073

[acel12646-bib-0178] Robin JD , Ludlow AT , Batten K , Magdinier F , Stadler G , Wagner KR , Shay JW , Wright WE (2014) Telomere position effect: regulation of gene expression with progressive telomere shortening over long distances. Genes Dev. 28, 2464–2476. 25403178 10.1101/gad.251041.114 PMC4233240

[acel12646-bib-0179] Robin JD , Ludlow AT , Batten K , Gaillard M‐C , Stadler G , Magdinier F , Wright WE , Shay JW (2015) SORBS2 transcription is activated by telomere position effect–over long distance upon telomere shortening in muscle cells from patients with facioscapulohumeral dystrophy. Genome Res. 25, 1781–1790. 26359233 10.1101/gr.190660.115 PMC4665000

[acel12646-bib-0180] Rodier F , Coppé J‐P , Patil CK , Hoeijmakers WAM , Muñoz DP , Raza SR , Freund A , Campeau E , Davalos AR , Campisi J (2009) Persistent DNA damage signalling triggers senescence‐associated inflammatory cytokine secretion. Nat. Cell Biol. 11, 973–979. 19597488 10.1038/ncb1909 PMC2743561

[acel12646-bib-0181] Rodriguez S , Coppedè F , Sagelius H , Eriksson M (2009) Increased expression of the Hutchinson‐Gilford progeria syndrome truncated lamin A transcript during cell aging. Eur. J. Hum. Genet. EJHG 17, 928–937. 19172989 10.1038/ejhg.2008.270 PMC2986496

[acel12646-bib-0182] Rodríguez SA , Grochová D , McKenna T , Borate B , Trivedi NS , Erdos MR , Eriksson M (2016) Global genome splicing analysis reveals an increased number of alternatively spliced genes with aging. Aging Cell 15, 267–278. 26685868 10.1111/acel.12433 PMC4783335

[acel12646-bib-0183] Rufini A , Tucci P , Celardo I , Melino G (2013) Senescence and aging: the critical roles of p53. Oncogene 32, 5129–5143. 23416979 10.1038/onc.2012.640

[acel12646-bib-0184] Sage J , Miller AL , Pérez‐Mancera PA , Wysocki JM , Jacks T (2003) Acute mutation of retinoblastoma gene function is sufficient for cell cycle re‐entry. Nature 424, 223–228. 12853964 10.1038/nature01764

[acel12646-bib-0185] Salminen A , Kauppinen A , Suuronen T , Kaarniranta K (2008) SIRT1 longevity factor suppresses NF‐κB ‐driven immune responses: regulation of aging via NF‐κB acetylation? BioEssays 30, 939–942. 18800364 10.1002/bies.20799

[acel12646-bib-0186] Salton M , Misteli T (2016) Small molecule modulators of pre‐mRNA splicing in cancer therapy. Trends Mol. Med. 22, 28–37. 26700537 10.1016/j.molmed.2015.11.005 PMC4707101

[acel12646-bib-0187] Sánchez‐Jiménez C , Ludeña MD , Izquierdo JM (2015) T‐cell intracellular antigens function as tumor suppressor genes. Cell Death Dis. 6, e1669. 25741594 10.1038/cddis.2015.43 PMC4385921

[acel12646-bib-0188] Scaffidi P , Misteli T (2006) Lamin A‐dependent nuclear defects in human aging. Science 312, 1059–1063. 16645051 10.1126/science.1127168 PMC1855250

[acel12646-bib-0189] Schmidt S , Essmann F , Cirstea IC , Kuck F , Thakur HC , Singh M , Kletke A , Jänicke RU , Wiek C , Hanenberg H , Ahmadian MR , Schulze‐Osthoff K , Nürnberg B , Piekorz RP (2010) The centrosome and mitotic spindle apparatus in cancer and senescence. Cell Cycle 9, 4469–4473. 21088502 10.4161/cc.9.22.13684

[acel12646-bib-0190] Schwerk C , Schulze‐Osthoff K (2005) Regulation of apoptosis by alternative pre‐mRNA splicing. Mol. Cell 19, 1–13. 15989960 10.1016/j.molcel.2005.05.026

[acel12646-bib-0191] Scott M , Bonnefin P , Vieyra D , Boisvert FM , Young D , Bazett‐Jones DP , Riabowol K (2001) UV‐induced binding of ING1 to PCNA regulates the induction of apoptosis. J. Cell Sci. 114, 3455–3462. 11682605 10.1242/jcs.114.19.3455

[acel12646-bib-0192] Scotti MM , Swanson MS (2015) RNA mis‐splicing in disease. Nat. Rev. Genet. 17, 19–32. 26593421 10.1038/nrg.2015.3 PMC5993438

[acel12646-bib-0193] Scrable H , Sasaki T , Maier B (2005) DeltaNp53 or p44: priming the p53 pump. Int. J. Biochem. Cell Biol. 37, 913–919. 15743665 10.1016/j.biocel.2004.11.014

[acel12646-bib-0194] Semba RD , Ferrucci L , Bartali B , Urpí‐Sarda M , Zamora‐Ros R , Sun K , Cherubini A , Bandinelli S , Andres‐Lacueva C (2014) Resveratrol levels and all‐cause mortality in older community‐dwelling adults. JAMA Intern. Med. 174, 1077. 24819981 10.1001/jamainternmed.2014.1582 PMC4346286

[acel12646-bib-0195] Serrano M , Lin AW , McCurrach ME , Beach D , Lowe SW (1997) Oncogenic ras provokes premature cell senescence associated with accumulation of p53 and p16(INK4a). Cell 88, 593–602. 9054499 10.1016/s0092-8674(00)81902-9

[acel12646-bib-0196] Sharpless NE , Sherr CJ (2015) Forging a signature of *in vivo* senescence. Nat. Rev. Cancer 15, 397–408. 26105537 10.1038/nrc3960

[acel12646-bib-0197] Shinozaki S , Chang K , Sakai M , Shimizu N , Yamada M , Tanaka T , Nakazawa H , Ichinose F , Yamada Y , Ishigami A , Ito H , Ouchi Y , Starr ME , Saito H , Shimokado K , Stamler JS , Kaneki M (2014) Inflammatory stimuli induce inhibitory S‐nitrosylation of the deacetylase SIRT1 to increase acetylation and activation of p53 and p65. Sci. Signal. 7, ra106. 25389371 10.1126/scisignal.2005375 PMC4340581

[acel12646-bib-0198] Shkreta L , Chabot B (2015) The RNA splicing response to DNA damage. Biomolecules 5, 2935–2977. 26529031 10.3390/biom5042935 PMC4693264

[acel12646-bib-0199] Shkreta L , Toutant J , Durand M , Manley JL , Chabot B (2016) SRSF10 connects DNA damage to the alternative splicing of transcripts encoding apoptosis, cell‐cycle control, and DNA repair factors. Cell Rep. 17, 1990–2003. 27851963 10.1016/j.celrep.2016.10.071 PMC5483951

[acel12646-bib-0200] Shkreta L , Blanchette M , Toutant J , Wilhelm E , Bell B , Story BA , Balachandran A , Cochrane A , Cheung PK , Harrigan PR , Grierson DS , Chabot B (2017) Modulation of the splicing regulatory function of SRSF10 by a novel compound that impairs HIV‐1 replication. Nucleic Acids Res. 45, 4051–4067. 27928057 10.1093/nar/gkw1223 PMC5397194

[acel12646-bib-0201] Shukla S , Kavak E , Gregory M , Imashimizu M , Shutinoski B , Kashlev M , Oberdoerffer P , Sandberg R , Oberdoerffer S (2011) CTCF‐promoted RNA polymerase II pausing links DNA methylation to splicing. Nature 479, 74–79. 21964334 10.1038/nature10442 PMC7398428

[acel12646-bib-0202] Sigala I , Ganidis G , Thysiadis S , Zografos AL , Giannakouros T , Sarli V , Nikolakaki E (2017) Lynamicin D an antimicrobial natural product affects splicing by inducing the expression of SR protein kinase 1. Bioorg. Med. Chem. 25, 1622–1629. 28139279 10.1016/j.bmc.2017.01.025

[acel12646-bib-0203] Sjögren K , Liu JL , Blad K , Skrtic S , Vidal O , Wallenius V , LeRoith D , Törnell J , Isaksson OG , Jansson JO , Ohlsson C (1999) Liver‐derived insulin‐like growth factor I (IGF‐I) is the principal source of IGF‐I in blood but is not required for postnatal body growth in mice. Proc. Natl Acad. Sci. USA 96, 7088–7092. 10359843 10.1073/pnas.96.12.7088 PMC22065

[acel12646-bib-0204] Skowyra D , Zeremski M , Neznanov N , Li M , Choi Y , Uesugi M , Hauser CA , Gu W , Gudkov AV , Qin J (2001) Differential association of products of alternative transcripts of the candidate tumor suppressor ING1 with the mSin3/HDAC1 transcriptional corepressor complex. J. Biol. Chem. 276, 8734–8739. 11118440 10.1074/jbc.M007664200

[acel12646-bib-0205] Smith PJ , Spurrell EL , Coakley J , Hinds CJ , Ross RJM , Krainer AR , Chew SL (2002) An exonic splicing enhancer in human IGF‐I pre‐mRNA mediates recognition of alternative exon 5 by the serine‐arginine protein splicing factor‐2/alternative splicing factor. Endocrinology 143, 146–154. 11751603 10.1210/endo.143.1.8598

[acel12646-bib-0206] Soliman MA , Berardi P , Pastyryeva S , Bonnefin P , Feng X , Colina A , Young D , Riabowol K (2008) ING1a expression increases during replicative senescence and induces a senescent phenotype. Aging Cell 7, 783–794. 18691180 10.1111/j.1474-9726.2008.00427.x

[acel12646-bib-0207] Southworth LK , Owen AB , Kim SK (2009) Aging mice show a decreasing correlation of gene expression within genetic modules G. P. Copenhaver, ed. PLoS Genet. 5, e1000776. 20019809 10.1371/journal.pgen.1000776 PMC2788246

[acel12646-bib-0208] Stavropoulou A , Halapas A , Sourla A , Philippou A , Papageorgiou E , Papalois A , Koutsilieris M (2009) IGF‐1 expression in infarcted myocardium and MGF E peptide actions in rat cardiomyocytes *in vitro* . Mol. Med. 15, 127–135. 19295919 10.2119/molmed.2009.00012 PMC2656994

[acel12646-bib-0209] Stilling RM , Benito E , Gertig M , Barth J , Capece V , Burkhardt S , Bonn S , Fischer A (2014) De‐regulation of gene expression and alternative splicing affects distinct cellular pathways in the aging hippocampus. Front. Cell. Neurosci. 8, 373. 25431548 10.3389/fncel.2014.00373 PMC4230043

[acel12646-bib-0210] Storer M , Mas A , Robert‐Moreno A , Pecoraro M , Ortells MC , Di Giacomo V , Yosef R , Pilpel N , Krizhanovsky V , Sharpe J , Keyes WM (2013) Senescence is a developmental mechanism that contributes to embryonic growth and patterning. Cell 155, 1119–1130. 24238961 10.1016/j.cell.2013.10.041

[acel12646-bib-0211] Takeuchi H , Rünger TM (2013) Longwave UV light induces the aging‐associated progerin. J. Invest. Dermatol. 133, 1857–1862. 23392295 10.1038/jid.2013.71

[acel12646-bib-0212] Tanahashi H , Tabira T (2001) Three novel alternatively spliced isoforms of the human beta‐site amyloid precursor protein cleaving enzyme (BACE) and their effect on amyloid beta‐peptide production. Neurosci. Lett. 307, 9–12. 11516562 10.1016/s0304-3940(01)01912-7

[acel12646-bib-0213] Tang Y , Horikawa I , Ajiro M , Robles AI , Fujita K , Mondal AM , Stauffer JK , Zheng Z‐M , Harris CC (2013) Downregulation of splicing factor SRSF3 induces p53β, an alternatively spliced isoform of p53 that promotes cellular senescence. Oncogene 32, 2792–2798. 22777358 10.1038/onc.2012.288 PMC6503963

[acel12646-bib-0214] Tissenbaum HA , Guarente L (2001) Increased dosage of a sir‐2 gene extends lifespan in *Caenorhabditis elegans* . Nature 410, 227–230. 11242085 10.1038/35065638

[acel12646-bib-0215] Tollervey JR , Wang Z , Hortobágyi T , Witten JT , Zarnack K , Kayikci M , Clark TA , Schweitzer AC , Rot G , Curk T , Zupan B , Rogelj B , Shaw CE , Ule J (2011) Analysis of alternative splicing associated with aging and neurodegeneration in the human brain. Genome Res. 21, 1572–1582. 21846794 10.1101/gr.122226.111 PMC3202275

[acel12646-bib-0216] Toyama T , Iwase H , Watson P , Muzik H , Saettler E , Magliocco A , DiFrancesco L , Forsyth P , Garkavtsev I , Kobayashi S , Riabowol K (1999) Suppression of ING1 expression in sporadic breast cancer. Oncogene 18, 5187–5193. 10498868 10.1038/sj.onc.1202905

[acel12646-bib-0217] Tucci P (2012) Caloric restriction: is mammalian life extension linked to p53? Aging 4, 525–534. 22983298 10.18632/aging.100481 PMC3461340

[acel12646-bib-0218] Varela I , Cadiñanos J , Pendás AM , Gutiérrez‐Fernández A , Folgueras AR , Sánchez LM , Zhou Z , Rodríguez FJ , Stewart CL , Vega JA , Tryggvason K , Freije JMP , López‐Otín C (2005) Accelerated ageing in mice deficient in Zmpste24 protease is linked to p53 signalling activation. Nature 437, 564–568. 16079796 10.1038/nature04019

[acel12646-bib-0219] Vaziri H , Dessain SK , Ng Eaton E , Imai SI , Frye RA , Pandita TK , Guarente L , Weinberg RA (2001) hSIR2(SIRT1) functions as an NAD‐dependent p53 deacetylase. Cell 107, 149–159. 11672523 10.1016/s0092-8674(01)00527-x

[acel12646-bib-0220] Vieyra D , Loewith R , Scott M , Bonnefin P , Boisvert F‐M , Cheema P , Pastyryeva S , Meijer M , Johnston RN , Bazett‐Jones DP , McMahon S , Cole MD , Young D , Riabowol K (2002a) Human ING1 proteins differentially regulate histone acetylation. J. Biol. Chem. 277, 29832–29839. 12015309 10.1074/jbc.M200197200

[acel12646-bib-0221] Vieyra D , Toyama T , Hara Y , Boland D , Johnston R , Riabowol K (2002b) ING1 isoforms differentially affect apoptosis in a cell age‐dependent manner. Cancer Res. 62, 4445–4452. 12154053

[acel12646-bib-0222] Vogelstein B , Lane D , Levine AJ (2000) Surfing the p53 network. Nature 408, 307–310. 11099028 10.1038/35042675

[acel12646-bib-0223] Von Zglinicki T (2002) Oxidative stress shortens telomeres. Trends Biochem. Sci. 27, 339–344. 12114022 10.1016/s0968-0004(02)02110-2

[acel12646-bib-0224] Wang E (1995) Senescent human fibroblasts resist programmed cell death, and failure to suppress bcl2 is involved. Cancer Res. 55, 2284–2292. 7757977

[acel12646-bib-0225] Wang ET , Sandberg R , Luo S , Khrebtukova I , Zhang L , Mayr C , Kingsmore SF , Schroth GP , Burge CB (2008) Alternative isoform regulation in human tissue transcriptomes. Nature 456, 470–476. 18978772 10.1038/nature07509 PMC2593745

[acel12646-bib-0226] Wang J , Geiger H , Rudolph KL (2011a) Immunoaging induced by hematopoietic stem cell aging. Curr. Opin. Immunol. 23, 532–536. 21872769 10.1016/j.coi.2011.05.004

[acel12646-bib-0227] Wang X , Zeng L , Wang J , Chau JFL , Lai KP , Jia D , Poonepalli A , Hande MP , Liu H , He G , He L , Li B (2011b) A positive role for c‐Abl in Atm and Atr activation in DNA damage response. Cell Death Differ. 18, 5–15. 20798688 10.1038/cdd.2010.106 PMC3131864

[acel12646-bib-0228] Werner H , Karnieli E , Rauscher FJ , LeRoith D (1996) Wild‐type and mutant p53 differentially regulate transcription of the insulin‐like growth factor I receptor gene. Proc. Natl Acad. Sci. USA 93, 8318–8323. 8710868 10.1073/pnas.93.16.8318 PMC38668

[acel12646-bib-0229] Whitaker R , Faulkner S , Miyokawa R , Burhenn L , Henriksen M , Wood JG , Helfand SL (2013) Increased expression of *Drosophila* sir2 extends life span in a dosedependent manner. Aging 5, 682–691. 24036492 10.18632/aging.100599 PMC3808700

[acel12646-bib-0230] Wood AM , Danielsen JMR , Lucas CA , Rice EL , Scalzo D , Shimi T , Goldman RD , Smith ED , Le Beau MM , Kosak ST (2014) TRF2 and lamin A/C interact to facilitate the functional organization of chromosome ends. Nat. Commun. 5, 5467. 25399868 10.1038/ncomms6467 PMC4235626

[acel12646-bib-0231] Yang SY , Goldspink G (2002) Different roles of the IGF‐I Ec peptide (MGF) and mature IGF‐I in myoblast proliferation and differentiation. FEBS Lett. 522, 156–160. 12095637 10.1016/s0014-5793(02)02918-6

[acel12646-bib-0232] Yeo GW , Coufal NG , Liang TY , Peng GE , Fu X‐D , Gage FH (2009) An RNA code for the FOX2 splicing regulator revealed by mapping RNA‐protein interactions in stem cells. Nat. Struct. Mol. Biol. 16, 130–137. 19136955 10.1038/nsmb.1545 PMC2735254

[acel12646-bib-0233] Yu C‐Y , Theusch E , Lo K , Mangravite LM , Naidoo D , Kutilova M , Medina MW (2014) HNRNPA1 regulates HMGCR alternative splicing and modulates cellular cholesterol metabolism. Hum. Mol. Genet. 23, 319–332. 24001602 10.1093/hmg/ddt422 PMC3869353

[acel12646-bib-0234] Zhang Z , Lotti F , Dittmar K , Younis I , Wan L , Kasim M , Dreyfuss G (2008) SMN deficiency causes tissue‐specific perturbations in the repertoire of snRNAs and widespread defects in splicing. Cell 133, 585–600. 18485868 10.1016/j.cell.2008.03.031 PMC2446403

[acel12646-bib-0235] Zhao W , Zhao J , Hou M , Wang Y , Zhang Y , Zhao X , Zhang C , Guo D (2014) HuR and TIA1/TIAL1 are involved in regulation of alternative splicing of SIRT1 pre‐mRNA. Int. J. Mol. Sci. 15, 2946–2958. 24566137 10.3390/ijms15022946 PMC3958892

[acel12646-bib-0236] Zheng H , Koo EH (2011) Biology and pathophysiology of the amyloid precursor protein. Mol. Neurodegener. 6, 27. 21527012 10.1186/1750-1326-6-27 PMC3098799

[acel12646-bib-0237] Zhu Y , Tchkonia T , Pirtskhalava T , Gower AC , Ding H , Giorgadze N , Palmer AK , Ikeno Y , Hubbard GB , Lenburg M , O'Hara SP , LaRusso NF , Miller JD , Roos CM , Verzosa GC , LeBrasseur NK , Wren JD , Farr JN , Khosla S , Stout MB , McGowan SJ , Fuhrmann‐Stroissnigg H , Gurkar AU , Zhao J , Colangelo D , Dorronsoro A , Ling YY , Barghouthy AS , Navarro DC , Sano T , Robbins PD , Niedernhofer LJ , Kirkland JL (2015) The Achilles' heel of senescent cells: from transcriptome to senolytic drugs. Aging Cell 14, 644–658. 25754370 10.1111/acel.12344 PMC4531078

[acel12646-bib-0238] Zohar O , Pick CG , Cavallaro S , Chapman J , Katzav A , Milman A , Alkon DL (2005) Age‐dependent differential expression of BACE splice variants in brain regions of tg2576 mice. Neurobiol. Aging 26, 1167–1175. 15917100 10.1016/j.neurobiolaging.2004.10.005

